# Computational design of an epitope-specific Keap1 binding antibody using hotspot residues grafting and CDR loop swapping

**DOI:** 10.1038/srep41306

**Published:** 2017-01-27

**Authors:** Xiaofeng Liu, Richard D. Taylor, Laura Griffin, Shu-Fen Coker, Ralph Adams, Tom Ceska, Jiye Shi, Alastair D. G. Lawson, Terry Baker

**Affiliations:** 1 UCB Celltech, 216 Bath Road, Slough, United Kingdom; 2 UCB Pharma, Chemin du Foriest 1, B-1420 Braine-l’Alleud, Belgium

## Abstract

Therapeutic and diagnostic applications of monoclonal antibodies often require careful selection of binders that recognize specific epitopes on the target molecule to exert a desired modulation of biological function. Here we present a proof-of-concept application for the rational design of an epitope-specific antibody binding with the target protein Keap1, by grafting pre-defined structural interaction patterns from the native binding partner protein, Nrf2, onto geometrically matched positions of a set of antibody scaffolds. The designed antibodies bind to Keap1 and block the Keap1-Nrf2 interaction in an epitope-specific way. One resulting antibody is further optimised to achieve low-nanomolar binding affinity by *in silico* redesign of the CDRH3 sequences. An X-ray co-crystal structure of one resulting design reveals that the actual binding orientation and interface with Keap1 is very close to the design model, despite an unexpected CDRH3 tilt and V_H_/V_L_ interface deviation, which indicates that the modelling precision may be improved by taking into account simultaneous CDR loops conformation and V_H_/V_L_ orientation optimisation upon antibody sequence change. Our study confirms that, given a pre-existing crystal structure of the target protein-protein interaction, hotspots grafting with CDR loop swapping is an attractive route to the rational design of an antibody targeting a pre-selected epitope.

Targeting the correct epitope is a critical step in selection of a monoclonal antibody to achieve the desired mechanism of action^1^. Current approaches for the discovery of novel antibodies for therapeutic and diagnostic use rely on raising antibodies against a target protein in immunised animals, or on *in vitro* selection of naïve or immunised libraries using display technologies. Both methods usually require extensive screening to meet the requirements of affinity, specificity, epitope and binding mode^2^,^3^. Attempts to engineer rationally antibodies have met with some success^4^,^5^. Computational antibody design has enabled rational engineering of antibodies to enhance affinity and stability by *in silico* mutation of interfacial CDR residues^6^,^7^, of which the application is largely limited to those antibodies with existing structures in complex with the corresponding antigen targets. Recent development of *de novo* antibody design approaches like OptMAVEn^8^ and AbDesign^9^ are based on protein-protein docking to sample the possible binding poses of artificial antibody scaffolds, followed by the generation of combinatorial backbone configurations and mutation design through exploiting sequence space of CDR loops, therefore theoretically enabling the targeting of a specific epitope by computational design. These methods have been virtually exemplified by design of antibody models that are close to the known antibodies’ sequences and reproduction of the crystal structure binding modes with the target proteins. Limited preliminary analysis of biological experiments has shown multiple, unique antibodies binding the tested antigen targets, albeit that the reported affinities are low, and that synthetic library approaches, like error-prone PCR or yeast display, are required to complete affinity maturation. Nevertheless, computational design of antibodies binding at pre-selected epitopes may complement sophisticated and efficient selection-based approaches, by accessing biologically relevant, conserved orthosteric sites, which may be immunogenic blind spots in sequences with extremely high homology between human and immunised animals^10^,^11^. The approach may also be relevant to the *de novo* design of agonistic antibodies and catalytic antibodies.

With very limited disclosure of successful examples of computational antibody design, especially lacking in structural validation of designed antibodies’ binding mode with antigen, the computational design of high-affinity antibodies targeting precise epitopes remains an elusive problem^12^. In this study, as a proof of concept, we applied a computational approach to demonstrate the rational design of an antibody binding at the pre-defined site of Keap1, a BTB-Kelch substrate adaptor protein that regulates steady-state levels of bZIP transcription factor Nrf2 in response to oxidative stress^13^. Although Keap1 is an intracellular drug target, that is not accessible to antibodies for therapeutic purposes, the Keap1-Nrf2 interaction features a concave binding surface surrounded by a six-blade β-propeller structure and readily identifiable hotspot residues (ETGE motif) from Nrf2 that serve as reference patterns to guide the design of novel antibodies targeting the Nrf2 binding site to block the cognate Keap1-Nrf2 interaction. This enables prioritisation of antibody scaffold binding configurations that present the desired binding patches on the antigen to the CDR loops, thus reducing the complexity of rational design of antibodies to block the Keap1-Nrf2 interaction by mimicking and displacing the binding partner Nrf2.

Antibody-Keap1 binding interfaces were created by grafting optimal orientations of hotspot residues from Nrf2 onto the geometrically compatible positions in CDR loops of a set of 1,417 antibody fragment variable region (Fv) scaffold crystal structures from Protein Data Bank (PDB), with the surrounding residue types and configurations further optimised to generate additional antibody–antigen interactions, while retaining original backbone conformations and V_H_/V_L_ pairing configurations. Several antibodies designed in this way showed low-micromolar binding affinity to Keap1. The affinities were further improved to nM level by *in silico* swapping of the CDRH3 loops across different antibody scaffold structures. Notably, the binding mode and interface with Keap1 of one antibody design were structurally validated by crystallography, illustrating that the antibody binds to Keap1 at Nrf2’s site in the manner as designed but with an unexpected domain drift observed in the V_H_/V_L_ pairing configuration. Although a set of pre-existing interaction patterns from the crystal structure of targeting protein-protein interaction are prerequisites at stage, the successful design of an epitope-specific antibody confirms computational approaches as a complementary route to selection-based methods for novel antibody discovery with precise control of the binding site that could be crucial for multiple applications. Also, comparing with direct use of the recombinant cognate protein binders as therapeutic agents to block the target protein-protein interactions, designing antibodies to precisely occlude the cognate protein’s binding site is more medically beneficial because of antibodies’ typically higher affinities and well-known Fc-mediated effector functionalities and long-acting efficacy in circulation^14^,^15^.

## Results

### Computational Design

**Figure 1:**
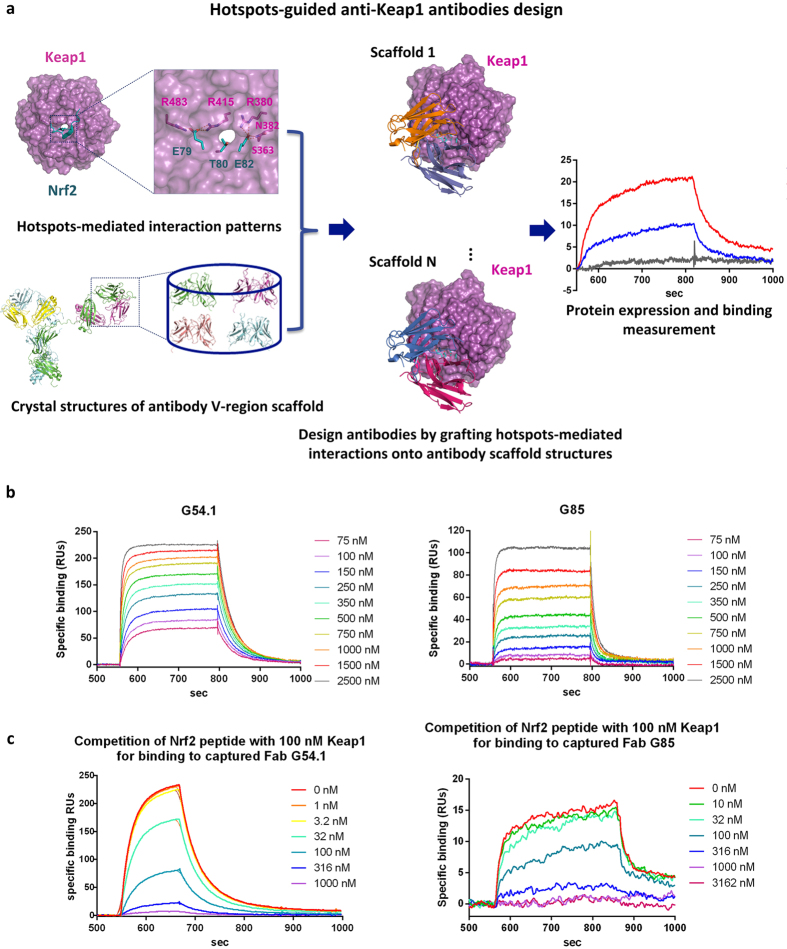
Design and characterisation of anti-Keap1 antibodies targeting Nrf2 binding site by hotspots graft. (**a**) Workflow scheme of antibody design using hotspots-guided scaffold graft approach. Three hotspots residues from Nrf2 are identified as the anchors, onto which the antibody Fv scaffold structures from PDB are aligned. The interfacial residues in CDR loops are mutated to reduce the clashes and improve the computed binding energies with Keap1. The designed models with different scaffolds hosting the hotspots are ranked by binding energies and the top rank ones are selected for synthesis and binding kinetics test using SPR. (**b**) SPR kinetics profiles for G54.1/Keap1 and G85/Keap1 complexes with designed antibody Fabs immobilized on the chips, which shows that these two example grafts bind to Keap1 and titrate well, with affinities of 126 nM and 236 nM respectively. (**c**) Competitive SPR kinetics profiles for G54.1/Keap1 and G85/Keap1 complexes by titration with cognate high affinity Nrf2 peptide segment, which shows that the two example grafts cross-block Nrf2 in a concentration-dependent manner as expected, indicating that the epitopes of the antibodies overlap the Nrf2 binding site on Keap1. The amount of G85 Fab was reduced to observe competition.

Inspired by the broadly validated strategy of hotspot-centric *de novo* design of protein binders^16^,^17^,^18^, we applied a computational method for designing novel antibodies binding at naturally occurring protein-binding protein interaction sites, guided by pre-defined hotspot-mediated interactions, either from cognate binders or from *in silico* hotspot residue placement approaches^19^,^20^. The computational workflow of antibody design targeting Keap1 is shown in Fig. 1a. Two hairpin loop motifs from Nrf2 (the stronger binding ETGE motif and the weaker binding DLG motif) have been reported to bind at the same region of two Keap1 Kelch domains^21^. There are conserved interactional patterns mediated by the crucial contact residues (hotspots) on both Nrf2 hairpin motifs, which are utilized to guide the design of Keap1-binding antibodies that are able to block Nrf2’s binding by occupying the same site on Keap1. By careful inspection of the binding interface of Keap1 and the higher affinity Nrf2 motif, three interactional patterns, derived from Nrf2 hotspot residues Glu79, Thr80 and Glu82 were revealed to be crucial in maintaining the strong electrostatic interactions and hydrogen bond networks with the adjacent Keap1 residues. The three hotspot residues were also identified using *in silico* alanine scanning (Supplementary Methods) to make sure that they were energetically important to the binding of Nrf2 and Keap1. We applied the hotspots-guided interface design approach with the aim of generating new interactions around the three hotspots based on backbone configurations of existing antibody CDR scaffolds. A residue-based triplet hashing method was implemented to search for the best antibody scaffolds from a collection of 1417 antibody Fv scaffold crystal structures as potential acceptors for the three Nrf2 hotspots, while maintaining the hotspots’ original interaction patterns with Keap1 (Methods). Five scaffolds that could host the hotspot residues in the correct relative orientations were selected and subjected to *in silico* mutagenesis to predict extra potential interfacial point mutations in CDR loops with improved binding energies to Keap1, leading to the generation of variants of original grafting designs.

### Experimental Characterization of Binding

Ten designed antibodies were expressed in the Fab format, and their binding affinities were measured by surface plasmon resonance (SPR). Eight of the ten selected antibody Fab designs showed detectable binding against Keap1, with the best two (G54.1 and G85) showing low micromolar binding affinities (Fig. 1b and Supplementary Table S1). Binding was reduced when a cognate Nrf2 peptide binder was added as a competitor (Fig. 1c), suggesting that the epitope of the designed antibodies on Keap1 may overlap with Nrf2’s site. Modelled structures showed that the three Nrf2 hotspots grafted onto CDRH2 loops of the two antibody scaffolds, along with CDRH1 and CDRH3 loops, presented similar conformations to the Nrf2 peptide and completely occupied the Nrf2 binding sites on Keap1 (Supplementary Fig. S1). Notably the original antibody scaffolds of G54.1 and G85 (PDB accession codes 3IVK and 2JB5, respectively) did not bind to Keap1, and none of the corresponding native antigens were biologically associated with Keap1 or Nrf2, strongly suggesting that the Keap1 binding of both antibodies was mediated via the computationally designed interfaces.

### Affinity Optimization

**Figure 2:**
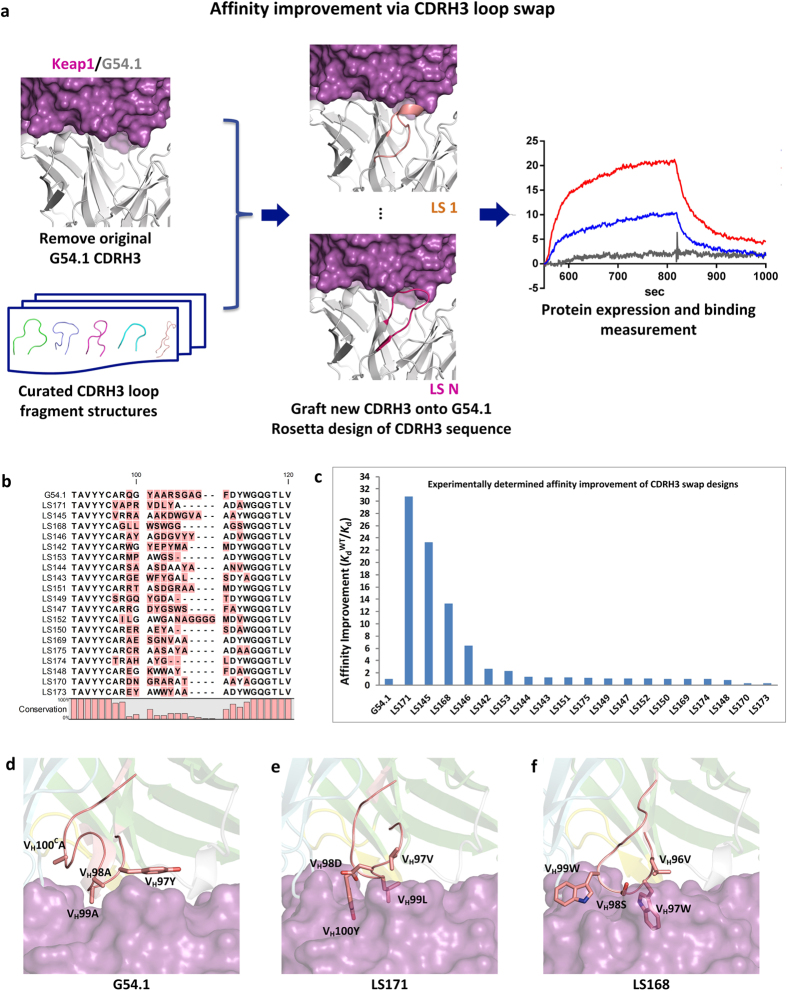
Design and characterisation of affinity improvement of G54.1 antibody by CDRH3 loop swap. (**a**) Workflow scheme of rational design of affinity improved G54.1 using CDRH3 loop swap approach. The original CDRH3 loop in the modelled Keap1-G54.1 structure was replaced by other CDRH3 fragments collected from PDB. The residues of CDRH3 in resulted new complex structures are subjected to computational design to improve the computed binding energies with Keap1. The top rank designs are selected for synthesis and binding kinetics test using SPR. (**b**) Sequence alignment of CDRH3 loop in the CDRH3 swap variants of G54.1 tested in this work, which shows low sequence identities between the CDRH3 loops of parental G54.1 and designed CDRH3 swap variants. (**c**) Experimentally determined binding affinity improvements of designed CDRH3-swap variants over parental G54.1 Fab. LS171, LS145, LS168 and LS146 show most prominent affinity improvement over G54.1, with the affinities of 4.1, 5.4, 9.5 and 19.6 nM respectively. (**d**) Computationally modelled CDRH3 conformations and interaction modes of G54.1, LS171 and LS168 with Keap1, highlighting the different loop conformations and interactions of the CDRH3 loops in the affinity-improved antibodies. The key contact residues in CDRH3 loops are depicted as sticks.

A barrier to designing high affinity antibodies is that current approaches treat their scaffolds as rigid structures with minimal perturbation of their backbone degrees of freedom. However, there is an experimentally validated precedent^22^,^23^ for transplanting CDR loops into different antibody frameworks due to the structural conservation of different loop types^24^, thus providing alternative, additional conformation degrees of freedom that have so far been untapped by rigid-scaffold design methods. Encoded by separated genes, CDRH3 is known as the most diverse antibody loop in terms of length and conformation among the six CDRs^25^,^26^,^27^. The modelled structure of Keap1 with G54.1 indicates that CDRH3 does not host any hotspot residues in G54.1 and was computationally predicted to contribute much less than CDRH2 to Keap1’s binding (Supplementary Fig. S2). In order to improve further the binding affinity, a computational design strategy was applied to swap the CDRH3 loop of G54.1 with ones from a curated CDRH3 loop fragment structure library (Fig. 2a and Methods), The CDRH3 sequences of generated chimeric Fv fragments in complex with Keap1 were further optimised using RosettaDesign^28^ and ranked by computed binding energy. Nineteen CDRH3-swap variants of G54.1 were chosen, expressed and purified (Fig. 2b), four of which show obviously improved affinities, with the best affinities of 4.1 and 5.4 nM measured from LS171 and LS145, representing respectively a 30- and 23-fold improvement of affinity over parental G54.1 (Fig. 2c and Supplementary Table S2), and rivalling the affinity of cognate Nrf2. LS148 and LS146, albeit with weaker affinities, show respectively 13- and 6-fold improvement. These four CDRH3 swap designs possessed completely new CDRH3 loops with different sequences, lengths, and conformations from G54.1, presenting improved shape complementarity scores with Keap1 (Supplementary Table S3). The affinity-improved G54.1 variants, as shown in the modelled structures (Fig. 2d–f), either utilise aromatic residue substitutions in shorter CDRH3 (10 vs. 13 of G54.1) to fill a void between G54.1 and the Keap1 surface (like V_H_99L and V_H_100Y in LS171, V_H_97W in LS168), or bear larger CDRH3 contact surface areas with Keap1 (like 2734 Å^2^ of LS168 vs. 2583 Å^2^ of G54.1), which may partly explain the improved bindings.

### Binding Mode Characterization

**Figure 3:**
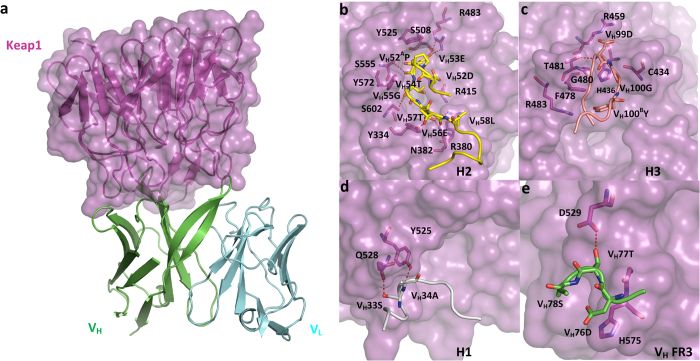
Crystal structure of LS146-scFv/Keap1 complex. (**a**) LS146-scFv /Keap1 crystal complex structure. **b-e,** Close-ups of interactions between Keap1 and individual loops in LS146-scFv: CDRH2 (**b**), CDRH3 (**c**), CDRH1 (**d**), and V_H_ framework 3 (FR3, **e**), respectively, with the key interactional residues depicted as sticks, and hydrogen bonds depicted as red dot lines. It shows that the interactions between antibody and Keap1 are dominantly mediated by heavy chain, and CDRH2 as the hotspots acceptor makes the most intense interactions.

We proceeded to crystallographic trials with the four highest-affinity CDRH3-swap designs in complex with Keap1, and for one of these, LS146, formatted as a single chain Fv (scFv), produced diffraction-quality crystals and a high resolution (1.85 Å) structure (Fig. 3a). Examination of this structure showed that LS146 bound almost exactly as designed in the Nrf2 binding site on Keap1 (Supplementary Fig. S3). CDRH2 adopts a similar conformation as that of the Nrf2 peptide and makes the most intensive contacts to Keap1 residues. The three hotspot residues are hosted at V_H_53E, V_H_54T, and V_H_56E within CDRH2, which mediates interactional hydrogen bond networks to Keap1 (involving R415, R483, S508, S602, R380, and N382), and there are several more interactions observed between CDRH2 and Keap1 residues including hydrogen bonds between V_H_57T with Y334 and N382, V_H_55G with S602 (Fig. 3b), rendering CDRH2 the most critical CDR loop to binding, corresponding well with the previous CDR loop binding contribution decomposition analysis. The 12-mer long CDRH3 loop folds into a hairpin-like conformation and interacts with the loops at the end of two Keap1 propeller blades as predicted (Fig. 3c). Other CDR loops involved in binding are CDRH1 (Fig. 3d) and part of V_H_ framework 3 (Fig. 3e).

**Figure 4:**
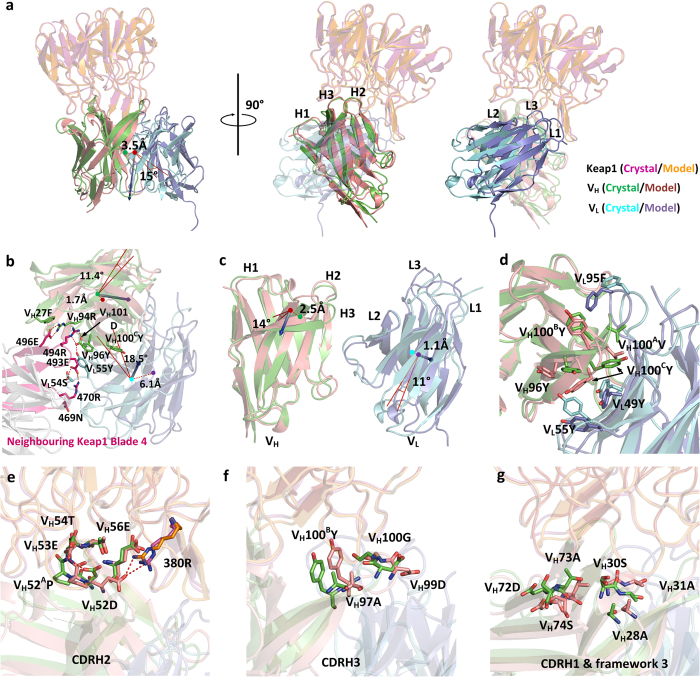
Comparison of LS146-scFv/Keap1 crystal complex structure with design model shows the precision of the computational design. (**a**) Comparison of binding modes of crystal LS146-scFv with modelled LS146-Fab by superimposing onto the Keap1 side, showing the antibody bind in the manner as modelled, though an obvious disengagement of V_L_ chain from the binding interface with Keap1 is observed. The coloured dots represent the centres of mass (COM) for crystal (green) and modelled (red) Fv structures; the blue vectors represent the axis around which the crystal structure rotates to superpose on the design model. The rigid body deviation between crystal and modelled Fv structures are characterised by the distances between the COMs and rotational angles. (**b**) Crystal packing between CDRH3, L2 loops and blade 4 of Keap1 from the neighbouring crystal unit, which leads to rigid distortion of both heavy and light chains. The key interactional residues of Keap1 from neighbouring crystal unit and the ones on the antibody side that undergo apparent conformational change from prediction are depicted as sticks. The respective rigid body deviations between crystal and modelled V_H_ and V_L_ domain are characterised by the distances between the COMs and rotational angles. (**c**) Individual rigid-body deviation of the CDRH3 loop (left) and V_L_ chain (right) by superposing the V_H_ chains of crystal structure on that of design model. The coloured dots represent the COMs of CDRH3 (left) and V_L_ (right); the blue vectors represent the axis around which the crystal structure rotates to superpose on the design model. The rigid body deviation between crystal and modelled structures are characterised by the distances between the COMs and rotational angles. (**d**) Close-up comparison of residues packing at V_H_/V_L_ interface from crystal and modelled structures when they are superposed on V_H_ chain. The key packing residues that undergo apparent conformational change from prediction are depicted as sticks. (**e–g**) Close-up comparison of backbone conformations and sidechain orientations of interfacial CDR loops from crystal and modelled structures: CDRH2 (**e**), CDRH3 (**f**), and CDRH1 and V_H_ framework 3 (**g**). The key contacting residues are depicted as sticks.

By superposing the crystal and design model structures on Keap1, the predicted LS146 conformation overlaid well with that of the crystal scFv in terms of general binding orientations and binding interface with Keap1 on the VH side, though there is an obvious unpredicted domain drift that disengages the V_L_ interactions completely from Keap1 (Fig. 4a). The whole Fv domain in the crystal structure is rotated by about 15° and drifted by 3.5 Å away from the design model, and V_L_ chain undergoes more obvious distortion than V_H_ chain. Superposing the crystal and modelled structures on the framework of V_H_ and V_L_ chain respectively shows that most frameworks and CDRs of V_H_ and V_L_ chain are nearly identical to the modelled structure (Supplementary Fig. S4), implying the deviation of the V_L_ interface with Keap1 between the crystal and predicted structures arises mainly from the change in V_H_/V_L_ orientation. Interestingly, this rigid-body deviation in Fv domain appears to be captured by the intensive hydrogen bonding network and sidechain stackings within the crystal packing between CDRH3, L2 loops and blade 4 of Keap1 from the neighbouring crystal unit (Fig. 4b). This unexpected V_H_/V_L_ orientation change was also characterised using the structural descriptors computed by ABangle^29^, and the most notable difference is observed in the V_H_/V_L_ torsional angle with a 10° deviation between the crystal and modelled structures, and detectable difference in terms of bend angles of LC1, HC2 and LC2, while both V_H_/V_L_ distance and other bend angles remain nearly identical (Table 1). However, both the V_H_/V_L_ torsional angles are very rarely observed among the antibody structures in the ABangle database with the same CDRH3 length (Supplementary Fig. S5). More detailed structural comparison shows that CDRH3 loop, though generally maintaining the hairpin-like conformation, undergoes an apparent rigid-body tilt of 14° and deviation of 2.5 Å away from the design model with the V_H_ chain framework superposed. The tilt of CDRH3 therefore shifts towards CDRL3 and further distorts the orientation of V_L_ chain by a rigid-body rotation of 11° and deviation of 1.1 Å from the design model (Fig. 4c). This CDRH3 tilt is likely caused by the sidechain flipping of V_H_100^C^Y towards the V_H_/V_L_ interface, leading to the loss of hydrophobic packing with V_L_49Y and sidechain reorganisation of V_H_96Y and V_L_55Y as well (Fig. 4d). It should be noted that the structure does not deviate from the designed key interfacial interactions to a significant degree, especially for the most important CDRH2 that makes the most intensive contacts with Keap1. The three hotspot residues on CDRH2 adopt nearly the same sidechain orientations as predicted with a full-atom RMSD of 1.6 Å, except for a flipped sidechain of V_H_52D due to an unexpected intramolecular hydrogen bond with the backbone amide of V_H_53E instead of the salt bridge with Keap1 380R as predicted (Fig. 4e). The other contacting residues on CDRH3, H1 and FR3 undergo bit more obvious deviation but still within vicinity of the contracting Keap1 residues (Fig. 4f and g).

## Discussion

In this study, we used a computational approach to design rationally orthosteric antibodies targeting the pre-defined epitopes exemplified by a proof-of-concept Keap1–Nrf2 interaction. This entails screening of the structurally best fitted antibody scaffolds to accommodate the known interaction patterns from the binding site of Nrf2, and designing mutations in the corresponding CDR sequences to improve the predicted binding energies with Keap1. This strategy is well-known as grafting-based design^30^ or heuristic negative design^31^, which introduces a binding site between two non-interacting proteins by grafting it from a different protein. Other state-of-art approaches like AbDesign and OptMAVEn rely on an alternative strategy known as dock-and-design by generating a conformer ensemble to represent the possible antigen binding poses, and optimising CDR sequences that are predicted to bind tightly with top ranked binding poses. The success of antigen positioning (or docking) is restricted by the accuracy of the sampling algorithm and scoring method, which in some cases struggle with prioritising the right conformations that present the desired binding patches on the antigen to the antibody scaffolds. Although such antibodies can interact through the designed residues, conformational changes at the interface may lead to reorientation of the binding pose or missing the targeted epitopes completely, as observed in the previous reported design of Prd-Pdar protein complex using dock-and-design strategy^32^. It therefore underscores the importance of encoding heuristic elements, like pre-defined interaction patterns from native binders for grafting into the suitable scaffolds, to ensure that the desired binding mode is favoured over alternatives. In our study, we attempted to entail this heuristic negative design strategy to emulate the conformational restriction of the desired binding site in prioritisation of binding poses. Although hotspots grafting method does not fully address the *de novo* antibody design challenge because the success is heavily dependent on the existence of prior structural knowledge of the cognitive binding partner interaction with the target protein for this approach to work, it provides an alternative to make full use of the existing knowledge and system-specific characteristics which may still not be fully captured in the calculations to improve the success rate. In the Keap1 case, hotspot residues come from the crystal co-complex structure with the cognate protein binder Nrf2, which may not always be available for every target of interest due to the lack of either biological or structural details. *In silico* hotspots placement approaches like MAPS^19^ or those using natural residue-like fragment MD simulation^20^ enable alternative strategies to steer grafting antibody scaffolds to the desired antigen epitopes independent of availability of cognate binders, therefore potentially expanding the application scope of this hotspots-based approach to targets with only unbound crystal structures.

Although this hotspots-guided grafting strategy has successfully led to the design of antibodies with detectable binding to Keap1 and cross-blocking the binding of Nrf2, the limitation is apparent, as shown in the previously reported design of non-antibody scaffold binders: the method still lacks the capability to build a high-affinity interface comparable to those of typical antibodies discovered from selection-based screening, without recourse to experimental affinity maturation. These observations highlight the challenges of antigen-antibody interface design which always involves large, polar binding surface-dominated loop interactions. One possible reason behind the inconsistency between predicted binding energies and experimental affinities is that the simplification of the underlying empirical energy functions, especially the employed Lazaridis-Karplus implicit solvation model^33^ which struggles with adequately characterising charged residue interactions with the solvent, and yields higher or similar water-to-protein transfer free energies for nonpolar as for many of the polar residues. Thus, the burial of unsaturated polar amino acids in the interface was wrongly favoured over nonpolar ones. Indeed, comparison of the computed properties of strong and weak binding designs suggests that more favourable binding energies, larger interfacial surface areas, and fewer buried unsaturated polar atoms contribute to higher binding affinities (Supplementary Table S3), despite no apparent correlations, corresponding with the adopted general empirical rules for design triage (Supplementary Methods). More sophisticated, physics-based scoring functions, AbDesign and OptMAVEn, apply site-specific amino acid probabilities to reduce the size of sequence optimisation spaces. These site-specific residue probabilities reflect known inter-atomic interactions and structural features to yield CDR sequence information consistent with naturally selected antibodies. The strategy taking advantage of pairwise residue interactional preference information from protein-protein interfaces has been utilised to engineer computationally the dengue virus (DV) stereotype selectivity of antibodies, leading to 450-fold affinity improvement against DV4^34^. Future work can consider incorporating this residue site-specific and pairwise interaction preference into mutation selection and design triage of the current antibody design approach.

Another possible restriction which led to a weak-affinity interface is the limited number of available scaffold structures in PDB. Although the hotspots can be locally accommodated on CDRH2 loop in some scaffolds, the other CDR loops may not be optimal in terms of both length and conformation to present the proper interactions around the grafted hotspots to the other epitope residues in Keap1. As with AbDesign and OptMAVEn, rational swapping of CDR configurations was utilized to enable exploration of alternative shapes and chemical complementarities that are untapped by using a limited number of antibody scaffold crystal structures. This is conceptually reminiscent of intrinsic V(D)J gene recombination during the early stages of B cell maturation leading to highly diverse antibody repertoire^35^, or *in vitro* synthetic display library constructed by CDR shuffling onto specified V-region frameworks to enlarge the diversity of synthetic antibody library^23^. The loop swap designs, with distinctively different CDRH3 backbone conformations and sequences from the original antibody scaffold used, show improved binding affinities without altering the epitope footprints.

The designed interactions of one of the CDRH3-swap antibodies with Keap1 were validated using X-ray crystallography, clearly confirming that the design recaptures the grafted interactional patterns from Nrf2 and creates the correct binding orientation and interactions around the hotspots residues within the pre-defined interface. Although the individual conformations of Keap1, V_H_ and V_L_ domains do not change considerably from the design model, there is an unpredicted apparent V_H_/V_L_ torsional angle change possibly led by a rigid-body tilt in CDRH3 loop, implying that precise antibody design exactly as modelled remains challenging because of the hard-to-predict CDRH3 loop conformation and structural correlation between V_H_/V_L_ orientation and antigen binding upon multiple mutated residues introduced in the CDR loops in the vicinity of the V_H_/V_L_ interface. Remodelling each CDRH3 loop backbone conformation and optimising the V_H_/V_L_ pairing orientation upon antibody sequence change during the design process may improve the accuracy of the design model^36^,^37^, but may be computationally unmanageable with the combinations of the design sequences and Fab scaffolds to be modelled using the approach in this study. On the other hand, the conversion from Fab to scFv has also been reported to cause variation in V_H_/V_L_ orientation and a subsequent reduction in affinity^38^. We observed that the potency of LS146-scFv is three-fold lower than, though still within an order of magnitude, that of its Fab form (Supplementary Fig. S6), implying the Fab-to-scFv conversion, along with CDRH3 tilt, may contribute to the local inconsistency between the crystal and model structures. Co-crystallography of Keap1 with LS146-Fab has been attempted as well, but no diffraction-quality crystal was obtained.

The antibodies designed here are not suitable for clinical development, as the Keap1–Nrf2 interaction is intracellular and not accessible by a therapeutic antibody. However, the Keap1-Nrf2 interaction features a concave binding surface in a six-blade β-propeller structure with readily identifiable hotspot residues that provide an ideal proof-of-concept system for structure-based design of novel antibodies targeting pre-selected epitopes to block the cognate protein-protein interactions orthosterically. Though CDRH2 of LS146 displays a similar β-strand structural configuration (Cα-atom RMSD = 0.27 Å) as well as high sequence identity (83%) with hotspot residue donor, the high-affinity Nrf2 ‘DEETGE’ peptide segment, which could lead to a speculation that insertion of Nrf2 loop in a CDR (e.g. H2, H3, or L1) of any antibody scaffold may confer binding, provided any steric clashes were avoided. However, this process may involve intensive analysis of all available antibodies’ sequences and structures to decide the best candidate CDR loops and insertion positions. A computationally heuristic strategy as reported here minimizes manual intervention and enables automatic selection of the most appropriate CDR scaffolds conformation and mutation positions for grafting all or part of the hotspot residues. It highlights the potential for designing antibodies targeting other protein-protein interactions in which either continuous or discontinuous heuristic patterns can be found on a cognate peptide binder to enable antibody scaffold grafting. Notably, low-density lipoprotein receptor-related protein (LRP5/6)-sclerostin (SOST) and LRP4-agrin represent other examples of a peptide binding into the hole of β-propeller^39^,^40^. The successful strategy described in this study, exemplified with Keap1, holds promise to transfer to the design of antibodies targeting these extracellular β-propeller proteins as well. Of course, it is arguable that all these cognate peptide segments binding with these β-propeller proteins as the hotspot residues donor feature the CDR-like β-strand structural configurations, making grafting compatible with the backbone of antibody CDR loops and therefore potentially precluding the application of the method to the design of antibodies by grafting the hotspot residues from native protein binders with alternative secondary structure types like helix bundles. But theoretically hotspots sidechains grafting is not restricted by backbone conformations or secondary structures of the hotspots, since individual hotspot sidechains can be geometrically fitted onto different CDR loops if the distance constraints encoded by the triplet hashing algorithm are identical between hotspots donor and antibody CDR loop acceptor positions, regardless of what secondary structure types these hotspot residues originally feature in the native donor proteins. Despite lacking a direct evidential case of our own, the reported successful design of helix-bundle proteins binding at antibody CR6261’s epitopes on hemagglutinin (HA) structure using the same hotspots grafting strategy implies the hotspots can be transferable between protein scaffolds featuring completely different secondary structures^16^, where the sidechain positions and backbone geometric alignments of two of the disembodied hotspot residues found through computational docking cluster into the regions contacting HA resemble those of the vicinal residues observed on the antibody CDRH2 and H3 loops respectively in the structure of the HA and CR6261 Fab complex.

Except for complementing selection-based methods to design novel antibodies for potential therapeutic and diagnostic usage for their recognised medical advantages over the other recombinant proteins, perhaps a more important niche where computational antibody design may have a role as biological probes that relate to the increasing use of antibodies as tools in membrane proteins (like G protein-coupled receptors, GPCRs) crystallography^41^ and in screening for small molecule inhibitors where in both cases the antibody traps a target molecule in a discrete conformational state that exposes a drug binding pocket^42^,^43^. Immunisation and panning routes to discover such antibodies may not be obvious, whereas rational design of an antibody that recognizes a transient conformation of a target protein, possibly predicted by molecular dynamics simulations, may provide an alternative route.

Overall, the Keap1 example presented here shows that computational design is now a viable tool to achieve new affinities and specificities of the existing antibody scaffolds, but that success is only partial and requires experimental validation. While still in its infancy, *in silico* design of antibodies holds great promise, and the methods described here demonstrate the potential. Computational design, to generate preliminary hit structures, may be complemented with focused phage library design and next generation sequencing^44^,^45^ to test, with great efficiency, permutations of further suggested amino acid substitutions to select second and subsequent round antibody structures with increased affinity, in an iterative cycle of computational refinement, selection and wet lab testing. With further improvements in computational accuracy and parallel optimisation of designed sequence space using modern oligonucleotide assembly methods, the structure-based computational design method offers a complementary method for rapid generation of antibodies to meet the diverse requirements from biology and healthcare research.

## Methods

### Computational methods

Full details for computational methods are given in Supplementary Methods. Anti-Keap1 antibodies targeting Nrf2 binding site were designed using a residue-based triplet hashing method to search for antibody scaffold crystal structures that are able to accommodate Nrf2 hotspots-mediated interaction patterns in the geometrically matched positions in CDRs, followed by CDRH3 swap to explore alternative loop configurations of the selected design. RosettaDesign was utilised to optimize the CDR loops’ sequences of the designs during these two stages to improve the predicted binding energy to Keap1. The pseudo codes for hotspots graft, CDRH3 swap, and RosettaScripts^46^ design protocols used are provided in Supplementary Data.

***Hotspots graft*** An in-house residue-based triplet hashing method^47^ was implemented to search for antibody scaffolds that were able to host hotspots-mediated interaction patterns from 1417 antibody crystal structures in SAbDab^48^, a database collecting and curating all the antibody structural data from PDB. A ‘triplet’ was defined as consisting of three virtual triangles that connected three residues’ backbone Cα, N and C atoms, respectively. Any three Nrf2 hotspots were compiled into a triplet and indexed with a unique key for looking up. All possible triplets of the CDRs residues in antibody scaffold structures were enumerated and indexed in the same way. The identical triplets from hotspots and antibody scaffolds were identified by comparing the respective index keys. The antibody scaffolds were grafted onto the hotspots by superimposing the scaffold triplet onto the corresponding identical hotspots one to minimise the RMSD between two sets of nine vertexes in the three triplet triangles. The three scaffold triplet residues were replaced with corresponding hotspots ones. The designed structures after triplet superimposition and hotspots graft were discarded if the backbone atoms of any residues in the grafted antibody scaffolds clashed with Keap1.

***CDRH3 loop swap*** This is based on the fact that both positions and orientations of backbone atoms of the CDRH3 root residues V_H_93 and V_H_103 in all Fab crystal structures are very conserved when they are superposed on V_H_ frameworks. Thus, the original orientation and conformation of an exogenous CDRH3 loop truncated from the other Fab crystal structure can be maintained, even with only V_H_93 and V_H_103 backbone atoms of the new CDRH3 superposed on the same set residues in the designed antibody framework. All the exogenous CDRH3 loops (V_H_93 to V_H_103) were dissected from the aforementioned 1417 antibody scaffold structures. The original CDRH3 loop of G54.1/Keap1 design model was removed from V_H_94 to V_H_102, leaving V_H_93 and V_H_103 as the anchor residues. Each exogenous CDRH3 loop was grafted by superposing the backbone atoms of its terminus V_H_93 and V_H_103 residues on the corresponding anchor residues of CDRH3-removed G54.1/Keap1 design model. Then the anchor residues were removed to enable ligating the grafted CDRH3 loop to G54.1 framework by connecting the new CDRH3 V_H_93 and V_H_103 with V_H_92 and V_H_104 of G54.1 framework, respectively. The new design structures were discarded if the backbone atoms of the new CDRH3 loop clashed with either G54.1 or Keap1.

***Rosetta sequence design*** Two rounds of sequence design were used, aiming for optimising the computed binding energies for the designs obtained from hotspots graft and CDRH3 loop swap, respectively. During the first round, starting from the five designed antibody structures that accommodated the three Nrf2 hotspots-mediated interaction patterns, each interfacial position in antibody side was singly mutated to all other amino acid types (excluding glycine, proline, and cysteine). Each mutation structure was optimized by repack and minimization of all the interfacial residues. The changes of computed binding energies for each point mutation (termed *ΔΔ*G) were evaluated in Rosetta full-atom scoring terms with the long-range electrostatics correction. Maximum five top ranked single point mutations in terms of lowest *ΔΔ*G scores were selected for manual incorporation into a combined mutant variant of each original design. During the second round, all CDRH3 residues in CDRH3-swap variants of G54.1 were allowed to mutate into all other amino acid types (excluding glycine, proline, and cysteine) simultaneously, with the backbone conformation of all interfacial residues on CDRs and Keap1 locally perturbed using backrub method, which has been reported to help improving mutant side-chains prediction^49^. Three iterations of sequence design were used to increase the likelihood that higher-affinity interactions could be found, starting with a soft-repulsive potential, and ending with the default standard van-der-Waals parameters.

***Design scoring*** Designs were evaluated by computed binding energy (Rosetta *Δ*G score), buried solvent accessible surface area (SASA), and shape complementarity (Sc) score^50^. High shape complementarity was enforced by rejecting designs with Sc < 0.5 in hotspots graft and Sc < 0.6 in CDRH3 swap. Rosetta total energy for each designed complex structure, and number of buried unsaturated polar atoms^51^ were used as the reference of the design quality evaluation as well (see Supplementary Methods).

### Binding analysis

Surface plasmon resonance (SPR) experiments were carried out on a Biacore 3000 system (GE Healthcare) and detailed experimental details are given in Supplementary Methods. Briefly, supernatant containing expressed Fab (or sham transfected supernatant control) was injected over immobilized anti-human F(ab’)_2_ polyclonal on a CM5 chip. A second injection of a Keap1 titration or a zero-analyte control allowed association and dissociation kinetics to be monitored. Chip regeneration completed each sensorgram cycle. Sensorgrams were corrected for baseline drift, caused by slow dissociation of captured Fab, by subtraction of an adjacent zero analyte control cycle. Non-specific binding of Keap1 at each concentration was corrected for by subtraction of the equivalent, baseline corrected, control supernatant cycle sensorgram. Biaevaluation™ software was used to fit association and dissociation kinetics and hence determine affinity constants (*K*_D_). Specificity of Fab binding to Keap1 was assessed by the same protocol by titration of an Nrf2 peptide analogue against a constant concentration of Keap1.

### Biochemical assays and structure determination

V_H_ and V_L_ genes of designed Fabs were expressed in HEK-293 cells using transcriptionally active PCR (TAP)^52^. For crystallographic analysis of LS146-scFv, a gene encoding V_H_ was fused to V_L_ through a (Gly_4_Ser)_4_ linker, with a carboxy-terminal tobacco etch virus (TEV) protease-cleavable His_10_ tag, and expressed in CHO-S XE cells. Detailed procedures for the Keap1 protein as well as antibodies expression, cloning, purification, crystallisation, and structure determination are given in Supplementary Methods. Atomic coordinates and structure factors for the reported crystal structure of LS146-scFv/Keap1 have been deposited with the Protein Data Bank under accession code 5F72.

## Additional Information

**Accession code**: Atomic coordinates and structure factors for the reported crystal structure of LS146-scFv/Keap1 have been deposited with the Protein Data Bank under accession code 5F72.

**How to cite this article:** Liu, X. *et al*. Computational design of an epitope-specific Keap1 binding antibody using hotspot residues grafting and CDR loop swapping. *Sci. Rep.* **7**, 41306; doi: 10.1038/srep41306 (2017).

**Publisher's note:** Springer Nature remains neutral with regard to jurisdictional claims in published maps and institutional affiliations.

## Supplementary Material

Supplementary Information

## Figures and Tables

**Table 1:** Structural V_H_/V_L_ orientation analysis using Abangle.

Structure	*HL*_torsion_(°)^1^	*HC1*_bend_(°)^2^	*HC2*_bend_(°)^3^	*LC1*_bend_(°)^4^	*LC2*_bend_(°)^5^	*dc*(Å)^6^
**LS146 design**	−56.50	71.59	118.94	123.50	79.80	16.06
**LS146 crystal**	−66.89	71.89	117.29	120.40	81.48	16.10

Two reference frame planes are mapped onto Fv structures. V_H_/V_L_ orientation is described as equivalent to measuring the orientation between the two planes by defining a vector ***C*** and three points on each plane as described in ref. 29.

^1^Torsion angle between *H1* and *L1*.

^2^Bend angle between *H1* and ***C***.

^3^Bend angle between *H2* and ***C***.

^4^Bend angle between *L1* and ***C***.

^5^Bend angle between *L2* and ***C***.

^6^Length of ***C***.
