# Supplementary material for: Computational design of an epitope-specific Keap1 binding antibody using hotspot residues grafting and CDR loop swapping
Source: Sci Rep. 2017 Jan 27;7:41306. doi: 10.1038/srep41306 (PMC5269676; doi:10.1038/srep41306)
Supplement: Supplementary Information [file srep41306-s1.pdf]

Computational design of an epitope-specific Keap1 binding antibody using hotspot residues grafting and CDR loop swapping

Xiaofeng Liu[a,*], Richard D. Taylor[a], Laura Griffin[a], Shu-Fen Coker[a], Ralph Adams[a], Tom Ceska[a], Jiye Shi[b], Alastair D.G. Lawson[a], and Terry Baker[a,*]

[a]UCB Celltech, 216 Bath Road, Slough, United Kingdom.

[b]UCB Pharma, Chemin du Foriest 1, B-1420 Braine-l'Alleud, Belgium.

*Corresponding authors

Supplementary Figures

Figure S1. a, The modelled binding poses of designed antibodies G54.1 (Left) and G85 (Right) in complex with Keap1; three hotspot residues (depicted as sticks) backbones on CDRH2 loops and Nrf2 peptide are superposed. **b,** *in vitro* SPR sensorgrams show original antibody Fab scaffolds of G54.1 and G85 do not bind with Keap1. The Fabs were immobilised on the chip by the anti-F(ab')2 antibody and titrated by Keap1 at 500nM concentration.

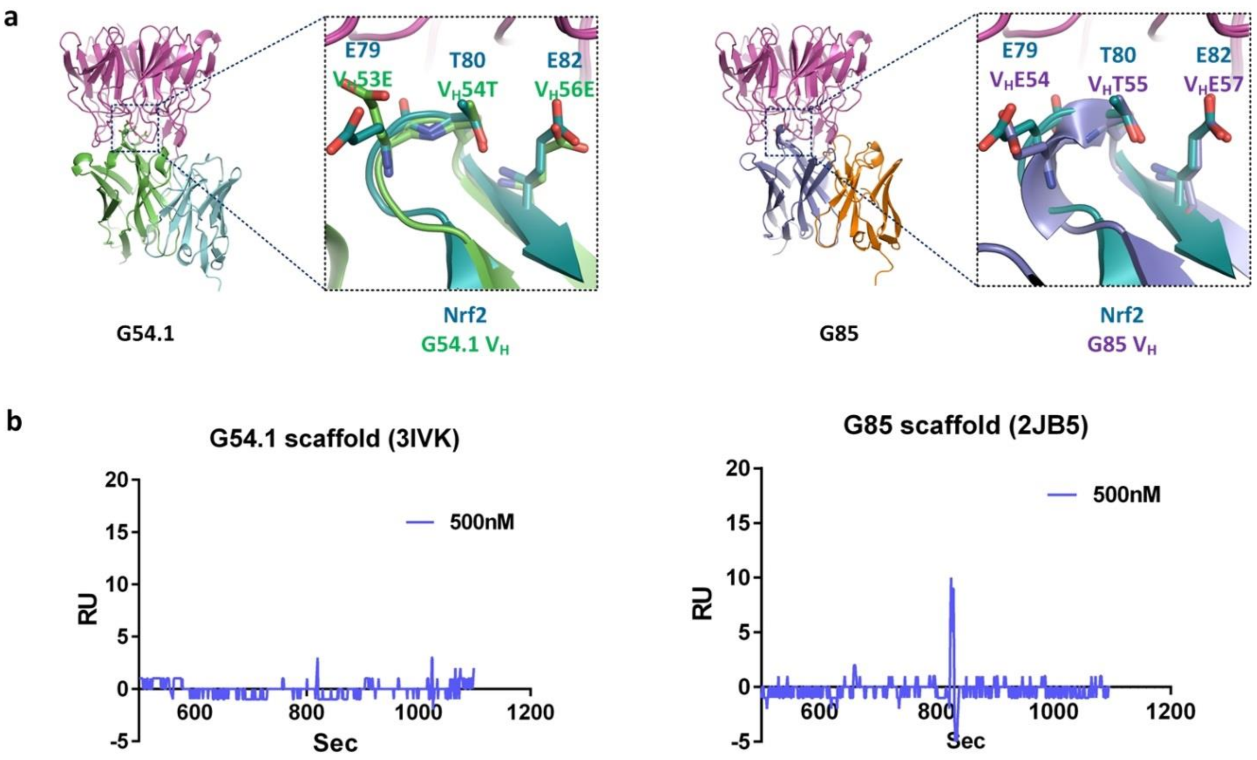

Figure S2. The modelled structure of Keap1 with G54.1 design indicates CDRH3 may be exchanged for affinity improvement. a, Predicted Keap1/G54.1 model structure. The six CDRs are highlighted, and the three grafted hotspots from Nrf2 in CDRH2 are shown as sticks. **b**, CDR-loop-wise Rosetta *Δ*G scores decomposition of designed G54.1 antibody. The individual CDR loop's contributions to the Rosetta *Δ*G scores between G54.1 and Keap1 were estimated by truncating each CDR loop from the Fv fragment of modelled G54.1/Keap1 complex structure.

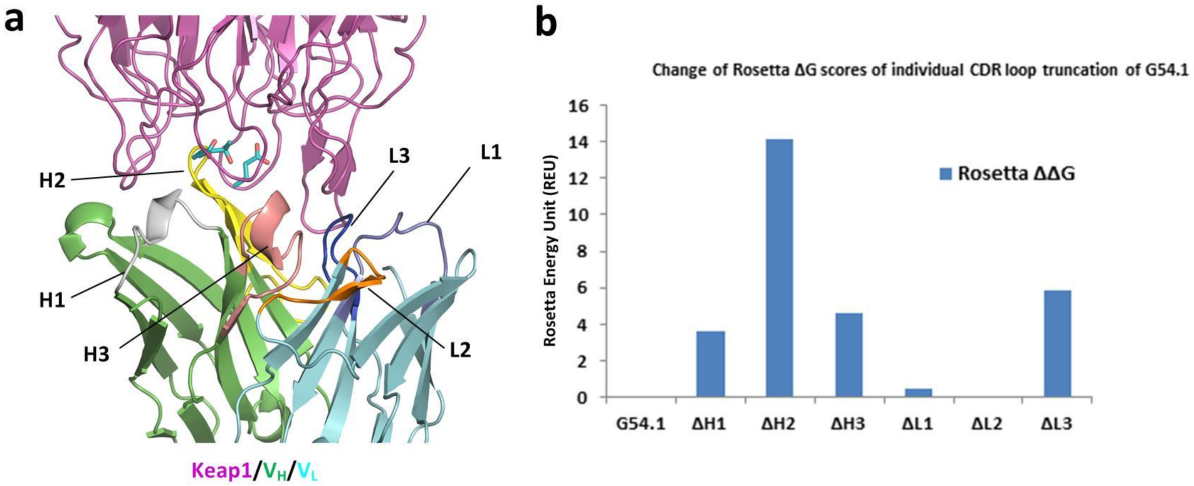

Figure S3. Binding sites of scFv-LS146 on Keap1. a, Crystal structure of LS146-scFv /Keap1 complex confirms occupation of Nrf2 binding site in Keap1. **b,** Close-up of LS146-scFv epitopes which are mapped onto Keap1 molecular surface coloured in terms of contacting CDRs.

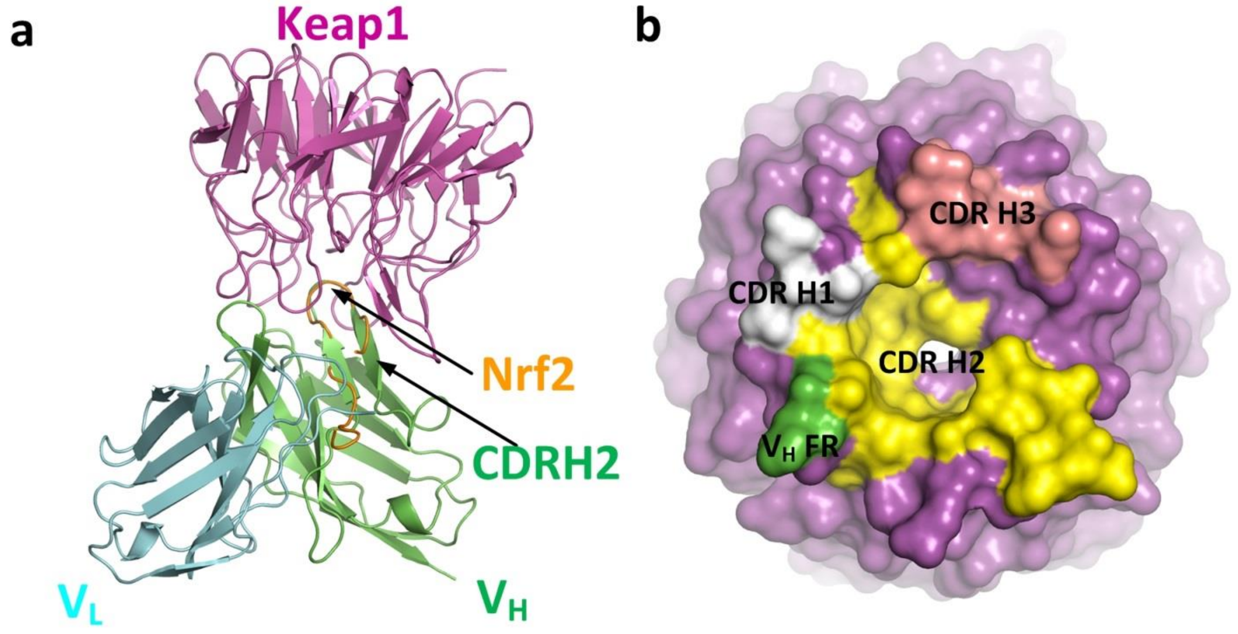

Figure S4. Comparison of V-region conformations of crystal and modelled structures of LS146. The structures are superposed on V_H (a) and V_L (b) chains, respectively.

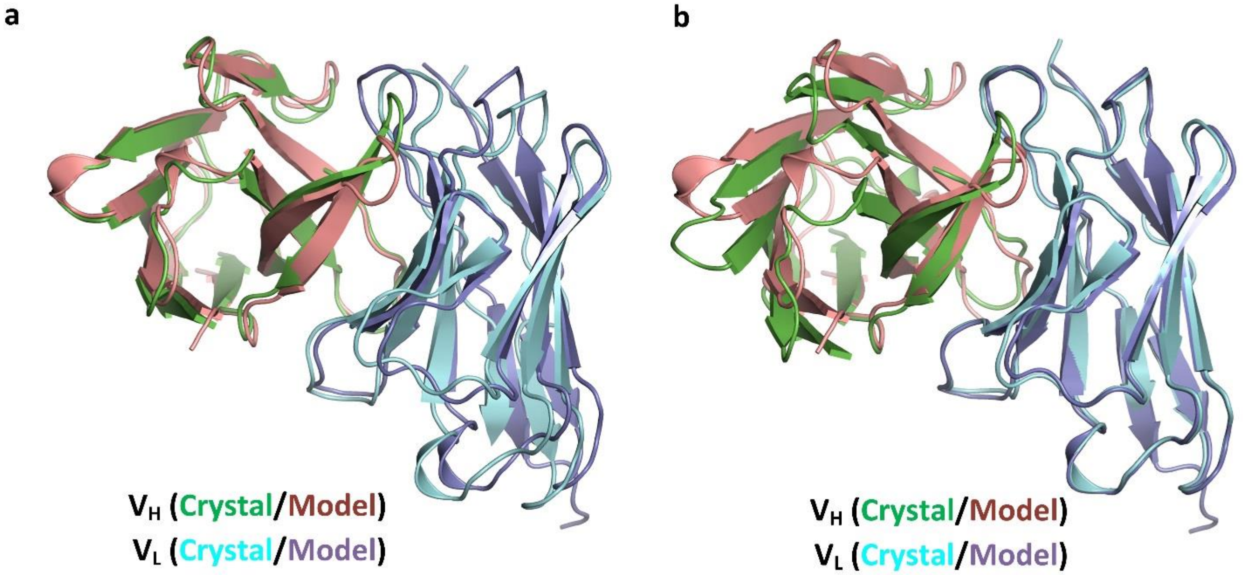

Figure S5. Histogram showing the distribution of V_H/V_L torsional angle for the non-redundant set of antibody structures in ABangle dataset with the same CDRH3 length to LS146. The crystal (red line) and modelled (green line) LS146 variable domains are placed at the corresponding positions in this structural space.

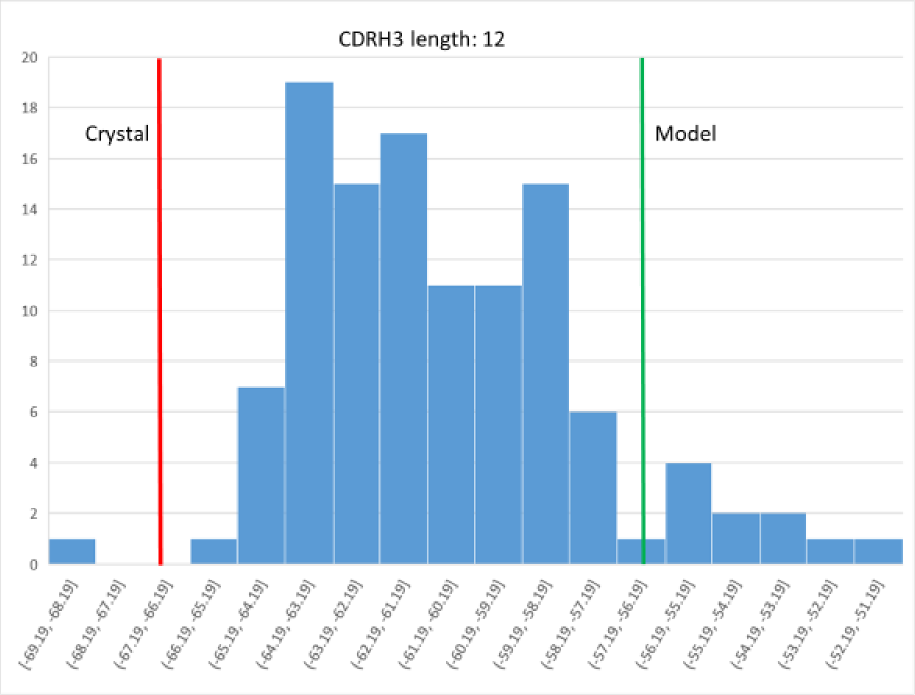

Figure S6. Comparison of potency of LS146-scFv versus -Fab in Biacore competition assay. IC_{50} values were calculated by fitting to the logarithm concentration versus normalized response/variable slope model: $Y = \frac{100}{1+10^{[(\log IC_{50} - X) \times S_{Hill}]}}$

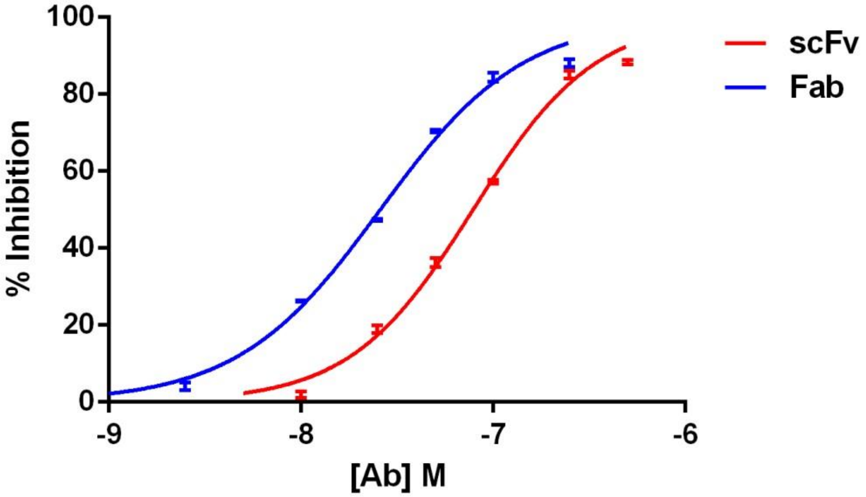

	IC50 nM	CI95%	
scFv	79	74 to 84	nM
Fab	26	24 to 28	nM

Supplementary Tables

Table S1. Binding affinities of the ordered antibody Fab designs from hotspots graft. Dissociation constants (K_D) were determined by SPR.

Design	Scaffold [1]	Hotspots positions	#Mutations from scaffolds (except grafted hotspots)	Fraction of Fab binding sites occupied @500nM Keap1[3]	k_{on} ($M^{-1} s^{-1}$)	k_{off} (s^{-1})	K_D (nM)	K_D 95% CI[4]
G53	2YSS[a]	V_H53E, V_H54T, V_H56E	3	0.002	ND[2]	ND	ND	ND
G53.1			5	0.0009	ND	ND	ND	ND
G54	3IVK[b]	V_H53E, V_H54T, V_H56E	6	0.01	ND	ND	ND	ND
G54.1			9	0.468	2.1×10^5	2.6×10^{-2}	126	110-143
G55	3TCL[c]	V_H102E, V_H102[A]T, V_H102[C]E	1	0.015	ND	ND	ND	ND
G55.1			3	0.016	ND	ND	ND	ND
G56	3U4B[d]	V_H102E, V_H102[A]T, V_H102[C]E	1	0.023	ND	ND	ND	ND
G56.1			2	0.027	ND	ND	ND	ND
G85	2JB5[e]	V_H54E, V_H55T, V_H57E	6	0.179	2.3×10^5	4.9×10^{-2}	236	137-405
G85.1			7	0.171	6.8×10^4	2.3×10^{-2}	341	209-555

[1] Original antigens in the PDB structures: [a] Hen Lysozyme; [b] RNA fragment; [c, d] HIV-1 Envelope Glycoprotein Gp120; [e] Diagnostic dye molecule.

[2] ND: Not determined.

[3] Limit if detection = 0.008

[4] 95% confidence intervals of K_D

Table S2. Computational features and binding kinetic data of ordered CDRH3-swap variants of G54.1. Dissociation constants (K_D) were determined by SPR.

Design	CDRH3 donor[1]	CDRH3 length	#Mutations from original CDRH3 donor	k_{on} (M^{-1} s^{-1})	k_{off} (s^{-1})	K_D (nM)	K_D 95% CI
171	2VDO	10	6	2.4×10^5	9.0×10^{-4}	4.1	3.2-5.3
145	2R0Z	13	8	2.1×10^5	1.1×10^{-3}	5.4	4.9-5.9
168	1ND0	10	2	2.7×10^5	2.5×10^{-3}	9.5	8.6-10.4
146	3DET	12	5	2.7×10^5	5.2×10^{-3}	19.6	18.6-20.5
142	1IGC	11	3	2.3×10^5	1.1×10^{-2}	47	45-50
153	4HWE	9	2	3.5×10^5	1.9×10^{-2}	54	50-58
144	2OSL	12	9	3.2×10^5	2.9×10^{-2}	93	80-107
143	1NCD	11	5	3.2×10^5	3.1×10^{-2}	99	83-118
151	3TT1	12	4	3.1×10^5	3.1×10^{-2}	103	95-111
175	3U9P	11	4	1.8×10^5	2.0×10^{-2}	110	87-139
149	3NTC	9	5	3.9×10^5	4.4×10^{-2}	113	105-122
147	3GK8	11	3	1.1×10^5	1.3×10^{-2}	119	98-143
152	3UJJ	15	2	8.6×10^4	1.0×10^{-2}	119	112-126
150	3SQO	9	3	3.4×10^5	4.1×10^{-2}	122	104-143
169	2ADG	11	4	2.0×10^5	2.4×10^{-2}	123	96-160
174	3E8U	8	3	2.8×10^5	3.3×10^{-2}	126	87-183
148	3KYK	10	5	4.5×10^5	7.1×10^{-2}	160	129-199
170	2V17	11	2	1.6×10^5	6.0×10^{-2}	393	294-524
173	3DVN	11	3	2.4×10^5	9.5×10^{-2}	413	283-601

[1] PDB antibody structures of the exogenous CDRH3 loops

Table S3. Computational features of experimentally tested antibody designs.

Design	Rosetta *ΔG* (REU)	Rosetta total energy (REU)	Buried SASA (Å^2)	Shape complementarity	Buried unsaturated polar atoms
G53	-14.6	-854.4	2276	0.59	15
G53.1	-20.8	-989.4	2175	0.57	14
G54	-16.8	-815.6	2514	0.61	9
G54.1	-32.3	-993.1	2583	0.58	4
G55	-15.6	-981.1	1453	0.57	3
G55.1	-19.7	-1089.8	1352	0.51	2
G56	-14.2	-973.7	1894	0.55	10
G56.1	-23.3	-1074.2	1650	0.53	3
G85	-15.8	-791.0	2624	0.59	19
G85.1	-19.5	-938.4	2706	0.56	19
LS171	-43.24	-1063.6	2590	0.63	14
LS145	-46.25	-1058.7	2734	0.65	10
LS168	-46.4	-1063.0	2656	0.64	15
LS146	-45.6	-1080.4	2663	0.63	12
LS142	-46.9	-1071.0	2628	0.65	8
LS153	-45.5	-1080.5	2548	0.65	9
LS144	-45.1	-1076.3	2618	0.67	10
LS143	-45.1	-1054.6	2643	0.65	11
LS151	-46.8	-1085.5	2557	0.65	13
LS149	-39.5	-1054.5	2615	0.6	5
LS147	-43.3	-1068.8	2512	0.64	7
LS152	-41.7	-1040.1	2497	0.66	12
LS150	-38.2	-1065.6	2507	0.62	8
LS169	-41.5	-1060.5	2429	0.63	9
LS175	-43.3	-1071.3	2588	0.64	9
LS174	-43.5	-1060.1	2335	0.67	10
LS148	-43.6	-1066.3	2645	0.66	9
LS170	-43.4	-1073.7	2498	0.67	10
LS173	-45.9	-1083.4	2680	0.61	10

Table S4. Crystallography data collection and structure refinement statistics of LS146-scFv/Keap1 complex.

**Data collection**	
Space group	$P2_1$
Cell dimensions	
a, b, c (Å)	70.5, 69.8, 99.6
α, β, γ (°)	90.0, 90.2, 90.0
Resolution (Å)	29.69-1.85
R_{sym} or R_{merge}	0.049
$I/\sigma I$	11.2
Completeness (%)	99.16%
Redundancy	3.1
**Refinement**	
Resolution (Å)	1.85
No. reflections	77790
$R_{work/}$ R_{free}	22.1/26.1
No. atoms	
Protein	7769
Ligand/ion	0
Water	564
B-factors	
Protein	25.75
Ligand/ion	N/A
Water	29.82
R.m.s deviations	
Bond lengths (Å)	0.013
Bond angles (°)	1.528

Table S5. Fv regions' amino acid sequences of tested antibody designs from hotspots graft.

Design	Sequence	
	V_H	V_L
G53	QVQLQESGPGLMKPSETLSLTCSVSGDSIAADYWSWIRKPPGKGLEYIGYVSETGETYYNPSLKSRVTISVDASKNRFSLKLNSVTAADTAVYYCARWDGDYWGQGILVTVSS	EIVMTQSPATLSVSPGERATLSCRASQSIGNNLHWYQQKPGQAPRLLIYYASQSISGIPARFSGSGSGTEFTLTISSLQSEDFAVYYCQQANSWPYTFGGGTKVEIK
G53.1	QVQLQESGPGLMKPSETLSLTCSVSGDSIAADYWSWIRKPPGKGLEYIGYVDETGETYYNPSLKRRVTISVDASKNRFSLKLNSVTAADTAVYYCARWDGDYWGQGILVTVSS	EIVMTQSPATLSVSPGERATLSCRASQSIGNNLHWYQQKPGQAPRLLIYYASQSISGIPARFSGSGSGTEFTLTISSLQSEDFAVYYCQQANSWPYTFGGGTKVEIK
G54	EVQLVESGGGLVQPGGSLRLSCAASGFAISASSIHWVRQAPGKGLEWVASISPETGETYYADSVAGRFTISADTSKNTAYLQMNSLRAEDTAVYYCARQGYAARSGAGFDYWGQGTLVTVSS	DIQMTQSPSSLSASVGDRVTITCRASQSVSSAVAWYQQKPGKAPKLLIYSASSLYSGVPSRFSGSRSGTDFTLTISSLQPEDFATYYCQQSYSFPSTFGQGTKVEIK
G54.1	EVQLVESGGGLVQPGGSLRLSCAASGFAISASSIHWVRQAPGKGLEWVASIDPETGETLYAKSVAGRFTISADTSKNTAYLQMNSLRAEDTAVYYCARQGYAARSGAGFDYWGQGTLVTVSS	DIQMTQSPSSLSASVGDRVTITCRASQSVSSAVAWYQQKPGKAPKLLIYSASSLYSGVPSRFSGSRSGTDFTLTISSLQPEDFATYYCQQSYSFPSTFGQGTKVEIK
G55	EVQLVESGGGLIRPGGSLRLSCKGSGFIFENFGFGWVRQGPGKGLEWVSGTNWNGGDSRYGDSVKGRFTISRDNSNNFVYLQMNSLRPEDTAIYYCARGTDYTIDETGERYQGSGTFWYFDVWGRGTLVTVSS	EIVLTQSPDTLSLSPGERATLSCRASQSVHSRYFAWYQHKPGQPPRLLIYGGSTRATGIPNRFSAGGSGTQFTLTVNRLEAEDFAVYYCQQYGASPYTFGQGTKVEIR
G55.1	EVQLVESGGGLIRPGGSLRLSCKGSGFIFENFGFGWVRQGPGKGLEWVSGTNWNGGDSRYGDSVKGRFTISRDNSNNFVYLQMNSLRPEDTAIYYCARGTDYTIDETGERYQGSGTFWYFDVWGRGTLVTVSS	EIVLTQSPDTLSLSPGERATLSCRASQRVHAKYFAWYQHKPGQPPRLLIYGGSTRATGIPNRFSAGGSGTQFTLTVNRLEAEDFAVYYCQQYGSSPYTFGQGTKVEIR
G56	EVQLVESGGGLIRPGGSLRLSCKGSGFIFENFGFGWVRQGPGKGLEWVSGTNWNGGDSRYGDSVKGRFTISRDNSNNFVYLQMNSLRPEDTAIYYCARGTDYTIDETGERAQGSGTFWYFDVWGRGTLVTVSS	EIVLTQSPATLSVSPGERATLSCRASQNVHPRYFAWYQQKRGQSPRLLIHSGSTRAAGIADRFSGGGSGMHFTLTITRVEPEDFAVYFCQQYGGSPYTFGQGTRVELR
G56.1	EVQLVESGGGLIRPGGSLRLSCKGSGFIFENFGFGWVRQGPGKGLEWVSGTNWNGGDSQYGDSVKGRFTISRDNSNNFVYLQMNSLRPEDTAIYYCARGTDYTIDETGERAQGSGTFWYFDVWGRGTLVTVSS	EIVLTQSPATLSVSPGERATLSCRASRNVHPRYFAWYQQKRGQSPRLLIHSGSTRAAGIADRFSGGGSGMHFTLTITRVEPEDFAVYFCQQYGGSPYTFGQGTRVELR
G85	QVQLVQSGAEVKKPGSSVKVSCKASGGTAAAYAINWVRQAPGQGLEWMGNIEPETGEANYAQKFAGRVTITADESTSTAYMELSSLRSEDTAVYYCARYFMSYKHLSDYWGQGTLVTVSS	DIALTQPASVSGSPGQSITISCTGTSSDVGSNNYVSWYQQHPGKAPKLMIYGGSNRPSGVSNRFSGSKSGNTASLTISGLQAEDEADYYCRSWDSAAAYSVFGGGTKLTVL
G85.1	QVQLVQSGAEVKKPGSSVKVSCKASGGTAAAYAINWVRQAPGQGLEWMGNIEPETGEAIYAQKFAGRVTITADESTSTAYMELSSLRSEDTAVYYCARYFMSYKHLSDYWGQGTLVTVSS	DIALTQPASVSGSPGQSITISCTGTSSDVGSNNYVSWYQQHPGKAPKLMIYGGSNRPSGVSNRFSGSKSGNTASLTISGLQAEDEADYYCRSWDSAAAYSVFGGGTKLTVL

Supplementary Methods

Computational design methodology. This method is an extension of the hotspot-centric *de novo* binding protein design approach. The interactions mediated by these hotspot residues often involve hydrogen bond networks, tight hydrophobic packing, and strong salt bridges, are therefore energetically favourable and evolutionarily conserved[1-3]. A geometric hashing method was developed to graft the hotspots, from either cognate protein binders or from *in silico* placements, into antibody scaffold crystal structures. Due to very limited number of available antibody Fv scaffold crystal structures, it is challenging to design high-affinity antibodies bearing CDRs that form optimal shape/electrostatic complementarity to the selected epitope on target proteins. CDRs swap leverages the large number of sequences and experimentally determined CDR configurations from other antibody scaffolds to construct new chimeric antibody models. The two strategies used in a tandem way may serve fast generation of high affinity antibodies targeting the selected binding site guided by the hotspots-mediated interaction patterns. Various RosettaScript XML script files were taken from 'rosetta_demo/public/rosetta_scripts' subdirectory of Rosetta[4] installation and customised to adapt to Keap1 antibody design example in this study.

Nrf2 hotspots identification. Three Nrf2 hotspot residues dominating the binding to Keap1 were identified using *in silico* alanine scanning script *AlaScan.xml*. The binding energy of Nrf2 and Keap1 in the complex structure (PDB accession code 2FLU[5]) was predicted by calculating the Rosetta total energy difference using default all-atom forcefield (score12 weights) between bound and unbound structures, referred as Rosetta *Δ*G scores hereafter. Each Nrf2 residue was *in silico* mutated into alanine, and the top ranked three Nrf2 residues (Glu79, Thr80, and Glu82) with the Rosetta *Δ*G scores decreased by at least 0.8 Rosetta energy unit (REU) after alanine mutation were confirmed as hotspots. The hotspots conformations were diversified by generation of inverse rotamers starting from their side chain atoms nearest to the Keap1 surface using the *InverseRotamers.xml* script[6]. Extra rotamer sampling (two half step standard deviations) was performed around all side chain torsion angles.

Antibody V-region scaffold structures. The antibody V-region scaffold structures with at least one paired V_H/V_L stored in PDB were extracted from SabDab (http://opig.stats.ox.ac.uk/webapps/sabdab) database in 2014. Only the structures solved by X-ray crystallography were used, including both Fab and scFv formats. If multiple crystal copies were available for the same antibody structure with different chain identifiers, only the first copy which appeared in the PDB file was kept. Only the Fv regions were kept from the Fab structures. Abnum[7] was used to renumber the residues in the Fv structures according to Chothia numbering scheme[8]. Any structures with broken polypeptide CDR loops were discarded. Finally 1417 antibody Fv scaffold structures were kept for hotspots graft design.

Graft Nrf2 hotspots onto antibody scaffold structures. An in-house residue-based triplet hashing method was implemented to search for the best antibody scaffold structures to accommodate the three Nrf2 hotspots onto, while maintaining the hotspots original interaction patterns with Keap1. We defined a 'residue triplet' as consisting of three virtual triangles that connected three residues' backbone Cα, N and C atoms, respectively. The triplet is characterised by nine vertexes ($V_{\alpha 1}$, $V_{\alpha 2}$, $V_{\alpha 3}$, V_{N1}, V_{N2}, V_{N3}, V_{C1},

V_{C2} and V_{C3}, corresponding to the positions of nine backbone C_α, N, and C atoms of the three residues consisting of the triplet) and nine edges ($E_{\alpha1}$, $E_{\alpha2}$, $E_{\alpha3}$, E_{N1}, E_{N2}, E_{N3}, E_{C1}, E_{C2} and E_{C3}, corresponding to the edges from the three triangles). On the hotspots side, any three inverse rotamers were enumerated from the three Nrf2 hotspot residues (Glu79, Thr80, and Glu82) and compiled into a residue triplet. Each triplet was canonicalized by ensuring that the longest and second longest C_α edges always corresponded to $E_{\alpha1}$ and $E_{\alpha2}$, respectively. Each triplet was indexed into a unique string key by concatenating six edges' round-off (RO) lengths in order. For example, for a given triplet with $E_{\alpha1}$=6.32, $E_{\alpha2}$=4.67, $E_{\alpha3}$=8.8, E_{N1}=4.3, E_{N2}=3.93, E_{N3}=7.21, E_{C1}=5.28, E_{C2}=5.4 and E_{C3}=9.82 the key is expressed as:

$$Key = Concatenate[RO(\mathrm{E})] = 6594475510 \quad (1)$$

All of the non-redundant index keys of hotspots' triplets were stored into a lookup table for fast access to corresponding hotspot triplet's information, including vertex residue types and atomic coordinates to facilitate later grafting onto the CDRs of antibody scaffold structures.

On the antibody scaffold side, any three CDR residues were enumerated and compiled into a triplet. The index key lookup table was generated in the same way as for hotspots triplet. To find the antibody scaffold structures which are able to accommodate the three hotspot residues in the geometrically matched positions in CDRs, the identical hotspots and antibody scaffold triplets were identified by directly comparing the respective index keys. The antibody scaffolds were grafted onto the hotspots by superimposing the scaffold triplet onto the corresponding identical hotspots one to minimise the RMSD between two sets of nine vertexes of the three triplet triangles. The three scaffold triplet residues were replaced with corresponding hotspots' ones by fitting the hotspots backbone atoms onto those of antibody triplet ones.

For each antibody designs obtained from hotspots graft, the sidechains of interfacial residues in antibody scaffolds clashing with Keap1 atoms were mutated into alanine to reduce clashes. The heavy-atom RMSD of the hotspots sidechain atoms before and after replacement was calculated. All residues were repacked and minimised using the *ppk.xml* script. Several filters described below were applied to triage the designs:

- The heavy-atom RMSD of the hotspots before and after replacement onto the antibody scaffold was smaller than 2.0 Å.
- The buried solvent accessible surface area (SASA) upon binding was greater than 1200 Å^2.
- Shape-complementarity (Sc) score was greater than 0.5.
- The Rosetta *Δ*G score (binding energy) was lower than 0.0 REU.

The surviving designs that passed the filtering rules were finally ranked by Rosetta *Δ*G scores.

CDRH3 loop swap. The individual CDR loop's contributions to the Rosetta *Δ*G scores of G54.1 were calculated by truncating each CDR loop from the Fv region of modelled G54.1/Keap1 complex structure. The Rosetta *Δ*G scores of each CDR truncation mutant were re-calculated. Individual CDR's contribution to binding was estimated by computing the Rosetta *Δ*G scores difference between each CDR truncation mutant and the original G54.1 antibody.

All the exogenous CDRH3 loops from the antibody scaffold crystal structures used in previous hotspots graft stage were dissected at the positions from V_H93 to V_H103 of Fv structures and labelled as the CDRH3 anchor residues. To graft an exogenous CDRH3 loop onto G54.1, the original CDRH3 loop of G54.1 was removed at the positions from V_H94 to V_H102, leaving V_H93 and V_H103 as the Fv anchor residues. Each exogenous CDRH3 loop was fitted onto the G54.1 Fv structure by superimposing the backbone atoms from two sets of anchor residues. The Fv anchor residues of G54.1 were later removed and the grafted exogenous CDRH3 loop was ligated onto G54.1 Fv by connecting the CDRH3 anchor residues with the neighbouring G54.1 residues (V_H92 and V_H104). The resulting structures were discarded if the backbone atoms of the new CDRH3 loop clashed with original G54.1/Keap1 complex structure. Any CDRH3 residue sidechains clashing with G54.1/Keap1 residues were mutated to alanine to reduce clashes. The final structures obtained from CDRH3 swap were repacked and minimised using Rosetta *ppk.xml* script as in Step 2 and ranked by Rosetta *Δ*G scores.

Rosetta sequence design. Two rounds of sequence design were performed to optimise the binding affinities of the designed antibodies from hotspots graft and CDRH3 swap, respectively.

During the first round, starting from the five designed antibody structures that accommodated the three Nrf2 hotspots-mediated Keap1 interaction patterns, each interfacial CDR residue in the antibody side was mutated into other amino acid types (except cysteine, glycine and proline) to probe the mutation effect on Rosetta *Δ*G scores in order to identify mutants that were potentially able to improve the computed binding energies of designed antibodies with Keap1. The *MutationScanPB.xml* script for computing change in binding free energy during *in silico* mutagenesis using the scoring function with the modified electrostatics scoring term[9] was used to generate the single point mutants list. The point mutations were ranked by calculating the change of Rosetta *Δ*G scores, or, between each mutant and corresponding wild type structures. The top ranked single point mutations were selected and combined (maximum 5 mutations) to generate a variant of the original antibody graft.

During the second round, all residues of the swapped CDRH3 loops on G54.1 were allowed to mutate into all other amino acid types (excluding glycine, proline, and cysteine) simultaneously, with the backbone conformation of all interfacial residues on CDRs and Keap1 locally perturbed using backrub method[10], using *flexbb-interfacedesign.xml* script. Explicit electrostatics was not used in the scoring function. Three iterations of redesign and minimization were used to increase the likelihood that higher-affinity interactions could be found, starting with a soft-repulsive potential (soft_rep weights), and ending with the default all-atom forcefield (score12 weights). Similar filter rules previously described for hotspots grafting designs were used to triage and rank the resulting CDRH3-swap designed structures:

- The buried SASA upon binding was greater than 2000 $Å^2$.
- The Rosetta *Δ*G score was lower than -20.0 REU.
- Sc score was greater than 0.6.

Design scoring. All the previously described computational features used for filtering or ranking the designs (**Table S2 & S3**) were calculated by Rosetta *InterfaceAnalyzer* application[11]:

- Rosetta *ΔG* score, or binding energy was defined as the difference between the total system energy in the bound and unbound states. In each state, interface residues were allowed to repack.
- Rosetta total energy of the modelled complex structures.
- Buried solvent accessible surface areas (SASAs) were defined as the difference between the total system SASAs in the bound and unbound states.
- Shape-complementarity (Sc) score of the modelled antibody/Keap1 complex structures.
- Buried unsaturated polar atoms.

Finally, 10 designs in 5 unique scaffolds after hotspots graft (**Table S1**) and 19 CDRH3-swap variants of G54.1 were chosen for experimental testing (**Table S2** and **Fig. 2b**).

Keap1 expression & purification. The gene encoding the Kelch domain of Keap1 was cloned into the expression vector pET-28a in frame with an N-terminal His tag and a TEV protease cleavage site. The amino acid sequence of the of the gene product is GSMGHAPKVGRLIYTAGGYFRQSLSYLEAYNPSDGTWLDLADLQVPRSGLAGCVVGGLLYAVGGRNNSPDGNTDSSA LDCYNPMTNQWSPCAPMSVPRNRIGVGVIDGHIYAVGGSHGCIHHNSVERYEPERDEWHLVAPMLTRRIGVGVAVL NRLLYAVGGFDGTNRLNSAECYYPERNEWRMITAMNTIRSGAGVCVLHNCIYAAGGYDGQDQLNSVERYDVETETW TFVAPMKHRRSALGITVHQGRIYVLGGYDGHTFLDSVECYDPDTDTWSEVTRMTSGRSGVGVAVTME. The construct was transformed into *E.Coli* strain BL21 (DE3), which was subsequently cultured in 2TY medium containing 25ug/ml kanamycin at 37 °C. Protein production was induced with 0.3 mM isopropyl β-D-1-thiogalactopyranoside (IPTG) at an O.D.600 of 4. Glycerol-based feed (50 mM MOPS, 1 mM MgSO4/MgCl2, 2 % glycerol) was added to the culture immediately after addition of IPTG, and the cultured was incubated further at 17 °C overnight. Cells were harvested by centrifugation and lysed in a buffer containing 50 mM Tris pH 8.5, 50 mM NaCl, 10% glycerol, 0.5% tritom-X100, 20 mM imidazole and sufficient amount of protease inhibitors (Roche). The lysate, pre-cleared by centrifugation, was filtered with a 0.2 µM filter and then mixed with Ni-NTA beads (Qiagen). The beads were washed with 50 mM Tris pH 8, 150 mM NaCl, 50 mM imidazole and 1 mM DTT before Keap1 was eluted with the former buffer supplemented with imidazole to a concentration of 250 mM. After the His tag was cut off, the sample was applied to a Ni-NTA (Qiagen) column to remove any Ni-binding contaminating proteins. The flow-through was collected and further purified by size exclusion (Superdex 75, GE Healthcare) and, if necessary, ion exchange (Mono Q, GE Healthcare) chromatography. The purified keap1 was concentrated and stored in 20 mM Tris pH 7.5 and 5 mM DTT at -80 °C.

Antibody cloning & expression. Heavy and light chain variable region genes designed *in silico* were chemically synthesized by DNA2.0, Inc. Transcriptionally active PCR (TAP) was employed to separately amplify the heavy and light chain variable regions and subsequently introduce DNA sequences encoding the hCMV promotor sequence, human γ1 C_H1 and C_κ (Km3 allotype) constant regions and poly(A) tail. The resultant constructs contained all of the required components for transient cellular expression. To generate Fab fragments for SPR analysis, HEK-293 cells were transiently transfected with TAP products using 293Fectin lipid transfection (Life Technologies, according to the manufacturer's instructions).

Crystallographic trials with the top four high affinity CDRH3-swap antibodies in Fab formats failed to yield diffraction-quality crystals in complex with Keap1. To convert LS146 from a Fab to a scFv construct, a gene encoding V_H fused to V_L through a $(Gly_4Ser)_4$ linker, a His_{10} tag along with a TEV protease cleavage site was synthesized and cloned into a UCB proprietary expression vector by DNA2.0, Inc. The amino acid sequence of the gene product is EVQLVESGGGLVQPGGSLRLSCAASGFAISASSIHWVRQAPGKCLEWVASIDPETGETLYAKSVAGRFTISADTSKNTAY LQMNSLRAEDTAVYYCARAYAGDGVYYADVWGQGTLVTVSSGGGGSGGGGSGGGGSGGGGSDIQMTQSPSSLSAS VGDRVTITCRASQSVSSAVAWYQQKPGKAPKLLIYSASSLYSGVPSRFSGSRSGTDFTLTISSLQPEDFATYYCQQSYSFPS TFGCGTKVEIKRTENLYFQGHHHHHHHHHH. CHO-S XE cells, a CHO-K1 derived cell line were transiently transfected with plasmid DNA using electroporation.[12] Cells were removed by centrifugation and scFv-TEV-His tagged protein was purified by IMAC. Supernatant was filtered with a 0.2uM filter and then loaded into a HisTrap excel column (GE healthcare). The column was washed with 50 mM Tris pH 8, 150 mM NaCl, 45 mM imidazole before the antibody was eluted with 50 mM Tris pH 8, 150 mM NaCl, 250 mM imidazole. After the His tag was removed, the sample was applied to the HisTrap excel column again to remove the Ni- binding contaminating proteins. The flow-through was collected and further purified by size exclusion (Superdex 75, GE Healthcare) chromatography. Purified antibody was concentrated, in 50 mM HEPES pH 7.5, 150 mM NaCl, 5% glycerol, and stored in aliquots at -80 °C until required.

Binding analysis. Surface plasmon resonance (SPR) experiments were carried out on a Biacore 3000 system (GE Healthcare) using reagents from the same manufacturer. Fabs were captured on the surface of CM5 sensor chips via affinity purified goat polyclonal $F(ab')_2$ fragment specific to anti-human $F(ab')_2$ (Jackson 109-006-097). The latter was immobilised to the activated carboxymethyl dextran surface via amine coupling as follows: a fresh mixture of 50 mM N-hydroxysuccimide and 200 mM 1-ethyl-3-(3-dimethylaminopropyl)-carbodiimide was injected for 5 minutes at a flow rate of 10 µl/min, followed by 50 µg/ml anti-human $F(ab')_2$ in 10 mM acetate pH 5.0 buffer for 5 min at the same flow rate. Finally the surface was deactivated with a 10 minute pulse of 1 M ethanolamine•HCl pH 8.5. Reference flow cell was on the chip was prepared by omitting the protein from the above procedure, thus in the following experiments sensorgrams were obtained as the response unit difference between anti-$F(ab')_2$ and reference flow cells. Initial binding of Keap1 to expressed Fabs was assessed by injecting 50 µl supernatant, diluted 1 in 5 in running buffer, over the reference and anti-$F(ab')_2$ flow cells at a flow rate of 10 µl/min, followed by a 150 µl injection of 0, 500 or 5000 nM Keap1 in running buffer at a flow rate of 30 µl/min. After the dissociation phase lasting at least 5 min the chip surface was regenerated with two 60 sec pulses of 40 mM HCl interspersed with a 30 sec pulse of 5 mM NaOH at the same flow rate. Association and dissociation kinetics of Keap1 binding to captured Fabs were determined by the same protocol over at least 8 values of the following concentrations: 75, 100, 150, 250, 350, 500, 750, 1000, 1500, 2500, 3500 and 5000 nM. Zero Keap1 controls were interspersed between the former cycles in order to correct for baseline drift and sham transfected supernatant was assessed at each Keap1 concentration in order to determine and correct for non-specific binding of Keap1. Specificity of Fab binding to Keap1 was assessed by competition with a high-affinity Nrf2 peptide analogue, biotin-PEG-LQLDEETGEFLPIQ-amide, corresponding to Nrf2 residues 74 to 87 that comprise the stronger Keap1 binding loop motif. Peptide Keap1 binding in the presence of peptide titrations to captured Fabs was

followed using the above protocol. Using BIAevaluation™ software all sensorgrams were first transformed by subtracting a zero Keap1 control cycle and the corresponding non-specific control cycle prior to fitting dissociation and association kinetics. Dissociation constants (K_D) were estimated as the logarithmic mean of values measured over at least 6 Keap1 concentrations. IC_{50} values were calculated using GraphPad Prism™ software by fitting to the log concentration versus normalized response/variable slope model represented by the following equation, where percent inhibition values for the three report points were treated as replicates at each concentration:

$$Y = \frac{100}{1+10^{[(\log IC_{50}-X)\times S_{Hill}]}} \tag{1}$$

Crystallisation. Keap1 was buffer exchanged to the storage buffer of LS146-scFv (50 mM HEPES pH 7.5, 150 mM NaCl and 5% glycerol) prior to complex formation. This removed DTT from Keap1 storage buffer and prevented it from breaking the disulphide bonds in the antibody. Keap1 was then mixed with LS146-scFv at a molar ratio of 1:1.5 and incubated at room temperature for 30 minutes. The complex was purified by size exclusion chromatography (Superdex 75™ 26/60, GE Healthcare) and concentrated to 5 mg/ml. Initial crystallisation trials, with 200 nl protein solution plus 200 nl reservoir solution (Qiagen) in sitting-drop vapor-diffusion format, produced crystals in two conditions. Reproduction and optimization of one of the hit crystallization conditions (0.2 M sodium acetate and 20% PEG3500), using seed crystals obtained from the initial screening, generated diffraction quality crystals. The crystals were cryoprotected in mother liquor, supplemented with PEG 3350 to 35% (w/v), and vitrified in liquid nitrogen prior to data collection.

Crystallographic data collection and processing. Datasets from crystals LS146-scFv/Keap1 complex was collected at the Diamond Light Source synchrotron facility (Didcot, United Kingdom) on beamline 104-1 at a wavelength of 0.917 Å. Molecular replacement was performed using program PHASER[13] in the CCP4 software suite[14,15] using Keap1 (PDB accession code 1X2J[16]), V_H and V_K frameworks without CDR loops (PDB accession code 3IVK[17]) as the models. The solvent content of the crystal was determined as 46.09% and there are two copied of complexes in an asymmetric unit. Solutions were found in three stages; positions of two copies of Keap1 were searched and obtained first, and then the two copies of heavy chains and the two light chains. Refinement and model building were carried out using Refmac5.4 (REFinement of MACromolecular structures)[18] and COOT (Crystallography Object-Oriented Toolkit)[19], respectively. The geometric quality of the final model was validated using Rampage[20], ProCheck[21], SFCheck[22], and the validation tools provided by the RCSB Protein Data Base. Data collection and refinement statistics for LS146-scFv/Keap1 is provided in **Table S4**.

Supplementary References

1. Ofran, Y. & Rost, B. Protein–protein interaction hotspots carved into sequences. *PLoS Comput. Biol.* **3**, e119 (2007).

2. Hu, Z., Ma, B., Wolfson, H. & Nussinov, R. Conservation of polar residues as hot spots at protein interfaces. *Proteins* **39**, 331–342 (2000).

3. Ma, B., Elkayam, T., Wolfson, H. & Nussinov, R. Protein-protein interactions: structurally conserved residues distinguish between binding sites and exposed protein surfaces. *Proc. Natl Acad. Sci. USA* **100**, 5772–5777 (2003).

4. Das, R., Baker, D. Macromolecular modeling with Rosetta. *Annu. Rev. Biochem.* **77**, 363–382 (2008).

5. Lo, S.C., Li, X., Henzl, M.T., Beamer, L.J. & Hannink, M. Structure of the Keap1:Nrf2 interface provides mechanistic insight into Nrf2 signalling. *Embo J.* **25**, 3605–3617 (2006).

6. Fleishman, S.J. et al. Computational design of proteins targeting the conserved stem region of influenza hemagglutinin. *Science* **332**, 816–821 (2011).

7. Abhinandan, K.R. & Martin, A.C. R. Analysis and improvements to Kabat and structurally correct numbering of antibody variable domains. *Mol. Immunol.* **45**, 3832–3839 (2008).

8. Al-Lazikani, B., Lesk, A.M. & Chothia, C. Standard conformations for the canonical structures of immunoglobulins. *J. Mol. Bio.* **273**, 927–948 (1997).

9. Whitehead, T.A. et al. Optimization of affinity, specificity and function of designed influenza inhibitors using deep sequencing. *Nat. Biotechnol.* **27**, 543–548 (2012).

10. Smith, C.A., Kortemme, T. Backrub-like backbone simulation recapitulates natural protein conformational variability and improves mutant side-chain prediction. *J. Mol. Biol.* **380**, 742–756 (2008).

11. Stranges, P.B. & Kuhlman, B. A comparison of successful and failed protein interface designs highlights the challenges of designing buried hydrogen bonds. *Protein Sci.* **22**, 74–82 (2013).

12. Cain, K. *et al.* A CHO cell line engineered to express XBP1 and ERO1-Lα has increased levels of transient protein expression. *Biotechnol. Prog.* **29**, 697-706 (2013).

13. McCoy, A.J. *et al. Phaser* crystallographic software. *J. Appl. Crystallogr.* **40**, 658–674 (2007).

14. Potterton, E., Briggs, P., Turkenburg, M., & Dodson, E. A graphical user interface to the *CCP4* program suite. *Acta Crystallogr. Sect. D* **59**, 1131–1137 (2003).

15. Winn, M.D. *et al.* Overview of the *CCP4* suite and current developments. *Acta Crystallogr. Sect. D* **67**, 235–242 (2011).

16. Padmanabhan, B. *et al.* Structural basis for defects of Keap1 activity provoked by its point mutations in lung cancer. *Mol. Cell* **3**, 689–700 (2006).

17. Shechner, D.M. *et al.* Crystal Structure of the Catalytic Core of an RNA-Polymerase Ribozyme. S*cience* **326**, 1271–1275 (2009).

18. Murshudov, G.N., Vagin, A.A. & Dodson, E.J. Refinement of macromolecular structures by the maximum-likelihood method. *Acta Cryst.* **D53**, 240–255 (1997).

19. Emsley, P. & Cowtan, K. *Coot*: model-building tools for molecular graphics. *Acta Crystallogr. Sect. D* **60**, 2126–2132 (2004).

20. Lovell, C. Structure validation by Calpha geometry: phi, psi and Cbeta deviation. *Proteins* **50**, 437–450 (2002).

21. Laskowski, R.A., MacArthur, M.W., Moss, D.S., & Thornton, J.M. PROCHECK: a program to check the stereochemical quality of protein structures. *J. Appl. Crystallogr.* **26**, 283–291 (1993).

22. Vaguine, A.A., Richelle, J., & Wodak, S.J. SFCHECK: a unified set of procedures for evaluating the quality of macromolecular structure-factor data and their agreement with the atomic model. *Acta Crystallogr. Sect. D* **55**, 191–205 (1999).

Supplementary Data

Pseudo codes of hotspots grafting onto antibody scaffold structures:

```
# Main function: iterate all antibody scaffold structures, do graftScaffoldOntoHotspots
DEF Main (String AntigenPDB, String HotspotsPDB, String ScaffoldsPath):

    # load antigen and hotspots
    Protein antigen = readPDB (AntigenPDB)
    Protein hotspots = readPDB (HotspotsPDB)

    # Iterate each template
    FOR scaffoldPDB IN ScaffoldsPath:
        Protein scaffold = readPDB (scaffoldPDB)

        # generate grafted complex structure
        Protein graft = graftScaffoldOntoHotspots (antigen, hotspots, scaffold)

        # dump the transplant structure
        dumpPDB (graft)

# FUNCTION graftScaffoldOntoHotspots: graft one antibody scaffold onto the hotspots
DEF graftScaffoldOntoHotspots (Protein Antigen, Protein Hotspots, Protein Scaffold):

    # Enumerate all hotspots triplets and store in hotspotsTripletList
    List hotspotsTripletList = []
    FOR r1, r2, r3 IN hotspots:

        Triplet hotspotsTriplet = setupTriplet (r1, r2, r3)
        hotspotsTripletList.append (hotspotsTriplet)

    # Enumerate all template CDR triplets and store in scaffoldTripletList
    List scaffoldTripletList = []
    FOR r1, r2, r3 IN scaffold's CDR residues:

        Triplet scaffoldTriplet = setupTriplet (r1, r2, r3)
        scaffoldTripletList.append (scaffoldTriplet)

    # iterate each pair of scaffoldTriplets and hotspotsTriplets, find the pair with identical key, and align
the corresponding triplets
    List SolutionList = []
    FOR hotspotsTriplet IN hotspotsTripletList:

        FOR scaffoldTriplet IN scaffoldTripletList:

            IF hotspotsTriplet.key == scaffoldTriplet.key:

                # Alignment and residue mutation
```

Align the antibody template onto the Hotspots by corresponding triplets using rms fitting
Replace the three template triplet residues with the corresponding hotspots

# Clashing check
Mutate any clashing residues on antibody with antigen's backbones to alanines

IF clashes remain after alanine mutation:
  Discard current Graft
ELSE:
  Append current Graft to the SolutionList

Sort SolutionList by ascending hotspots RMSD

# Output the complex structure of antigen and transplanted antibody scaffold (with mutated Hotpots)
Return SolutionList.top

# CLASS Triplet and FUNCTION setupTriplet: Setup residue triplets
CLASS Triplet:
  Residue residue1, residue2, residue3
  String key

DEF setupTriplet (**Residue** r1, **Residue** r2, **Residue** r3):

  # Edge lengths of the resdue triangle by residue1.Cα, residue2.Cα, residue3.Cα
  $dC_\alpha12$ = **Distance** (r1.C_α, r2.C_α), $dC_\alpha23$ = **Distance** (r2.C_α, r3.C_α), $dC_\alpha13$ = **Distance** (r1.C_α, r3.C_α)

  # Edge lengths of the resdue triangle by residue1.N, residue2.N, residue3.N
  dN12 = **Distance** (r1.N, r2.N), dN23 = **Distance** (r2.N, r3.N), dN13 = **Distance** (r1.N, r3.N)

  # Edge lengths of the resdue triangle by residue1.C, residue2.C, residue3.C
  dC12 = **Distance** (r1.C, r2.C), dC23 = **Distance** (r2.C, r3.C), dC13 = **Distance** (r1.C, r3.C)

  # Filter the triangles with any length less than 3.5 A
  IF any dC_α, dN, or dC **<=** 3.5:
    Return False

  # r1 and r2 corresponds to the longest Cα edge, r1 and r3 corresponds to the shortest Cα edge
  Reorder r1, r2, r3 corresponding to descending $dC_\alpha12$, $dC_\alpha23$, $dC_\alpha13$

  # Indexing key of the triplets by rounding up the edge lengths and concatenating into string
  key = **String** (**roundup** ($dC_\alpha1$)) + **String** (**roundup** ($dC_\alpha2$)) + **String** (**roundup** ($dC_\alpha3$)) + **String** (**roundup** (dN1)) + **String** (**roundup** (dN2)) + **String** (**roundup** (dN3)) + **String** (**roundup** (dC1)) + **String** (**roundup** (dC2)) + **String** (**roundup** (dC3))

  # return reordered r1, r2, r3 and key into a triplet
  Return **Triplet** (r1, r2, r3, key)

Pseudo codes of CDRH3 loop swapping of G54.1:

# Main function: iterate all antibody CDRH3 loop structures, do swapCDRH3
DEF Main (**String** AntibodyAntigenComplexPDB, **String** CDRH3sPath):

# load antibody-antigen complex PDB structure
Protein system = **readPDB** (AntibodyAntigenComplexPDB)
# chop off wt CDRH3 loop
Protein truncatedH3System = **chopCDRH3** (system)

# Iterate each exogenous CDRH3 loop structure
FOR CDRH3LoopPDB **IN** CDRH3sPath:
Protein h3loop = **readPDB** (CDRH3LoopPDB)

# generate H3 swapped complex structure
Protein loopswap = **swapCDRH3** (truncatedH3System, h3loop)

# dump the transplant structure
dumpPDB (loopswap)

# FUNCTION swapCDRH3: graft one exogenous CDRH3 loop onto the CDRH3-truncated antibody-antigen complex structure
DEF swapCDRH3 (**Protein** truncatedH3System, **Protein** h3loop):

# Alignment of the anchor residues of exogenous H3 loop onto those of CDRH3-truncated Fv
Align the h3loop anchor residues (V_H93 and V_H103) onto those of truncatedH3System
Remove the original V_H93 and V_H103 residues from truncatedH3System
Ligate the backbones of new h3loop's V_H93 and V_H103 with V_H92 and V_H104 of truncatedH3System, respectively, generating a swappedH3System (new H3 loop inserted into original Fv in complex with antigen)

# Clashing check
FOR any clashing residues on h3loop with rest of swappedH3System's backbones, mutate them to alanine

IF clashes remain after alanine mutation:
Discard current swappedH3System
ELSE:
# Output the complex structure of antigen-Fv and transplanted new H3 loop
Return current swappedH3System

RosettaScripts: AlaScan.xml:

<dock_design>

```
	<FILTERS>
		<AlaScan name=scan partner1=0 partner2=1 scorefxn=score12
interface_distance_cutoff=8.0 repeats=3/>
	</FILTERS>
	<MOVERS>
		<RepackMinimize name=intermin scorefxn_repack=score12 scorefxn_minimize=score12
interface_cutoff_distance=8.0 repack_partner1=0 repack_partner2=0 design_partner1=0
design_partner2=0 minimize_bb=0 minimize_sc=1 minimize_rb=0/>
	</MOVERS>
	<PROTOCOLS>
		<Add mover_name=intermin/>
		<Add filter_name=scan/>
	</PROTOCOLS>
</dock_design>
```

RosettaScripts: InverseRotamers.xml:

```
<dock_design>
	<FILTERS>
		<EnergyPerResidue name=energy pdb_num=79B energy_cutoff=0.0/>
		<Ddg name=ddg threshold=-1.0/>
	</FILTERS>
	<MOVERS>
		<TryRotamers name=try pdb_num=79B/>
	</MOVERS>
	<PROTOCOLS>
		<Add mover_name=try/>
		<Add filter_name=energy/>
		<Add filter_name=ddg/>
	</PROTOCOLS>
</dock_design>
```

RosettaScripts: ppk.xml:

```
<dock_design>
	<MOVERS>
		<Prepack name=ppk jump_number=0 scorefxn=score12/> Jump_number=0 to prepack
	the entire structure without moving the partners apart.
		<MinMover name=min scorefxn=score12 chi=1 bb=0 jump=0/>
	</MOVERS>
	<PROTOCOLS>
		<Add mover_name=ppk/>
		<Add mover_name=min/>
	</PROTOCOLS>
</dock_design>
```

RosettaScripts: MutationScanPB.xml:

```
<dock_design>
	<SCOREFXNS>
		<local_score weights=score12_full patch="pb_elec.wts_patch"/>
		<local_score_soft weights=soft_rep patch="pb_elec.wts_patch"/>
	</SCOREFXNS>
	<TASKOPERATIONS>
		<InitializeFromCommandline name=init/>
		<ProteinInterfaceDesign name=pid repack_chain1=1 repack_chain2=1 design_chain1=0
design_chain2=1 interface_distance_cutoff=8/>
		<ProteinInterfaceDesign name=pio repack_chain1=1 repack_chain2=1 design_chain1=0
design_chain2=0 interface_distance_cutoff=8/>
	</TASKOPERATIONS>
	<MOVERS>
		<AtomTree name=docking_tree docking_ft=1/>
		<MinMover name=min_sc scorefxn=local_score bb=0 chi=1 jump=1/> minimize sc, rb
		<PackRotamersMover name=pack_interface scorefxn=local_score
task_operations=init,pio/>
		<PackRotamersMover name=pack_interface_soft scorefxn=local_score_soft
task_operations=init,pio/>
		<ParsedProtocol name=relax_before_baseline>
			<Add mover=docking_tree/>
			<Add mover=pack_interface/>
			<Add mover= min_sc/>
		</ParsedProtocol>
	</MOVERS>
	<FILTERS>
		<Ddg name=ddg scorefxn=local_score confidence=0.0/>
		<Delta name=delta_ddg filter=ddg upper=1 lower=0 range=-0.5
relax_mover=relax_before_baseline/>
		<FilterScan name=scan_binding scorefxn=local_score
relax_mover=relax_before_baseline task_operations=pid,init filter=delta_ddg triage_filter=delta_ddg
resfile_name="scan.resfile"/>
		<Time name=scan_binding_timer/>
	</FILTERS>
	<PROTOCOLS>
		<Add mover=docking_tree/>
		<Add filter=scan_binding_timer/>
		<Add filter=scan_binding/>
		<Add filter=scan_binding_timer/>
	</PROTOCOLS>
</dock_design>
```

RosettaScripts: FlexbbInterfaceDesign.xml:

```
<dock_design>
	<TASKOPERATIONS>
		<ProteinInterfaceDesign name=pio repack_chain1=1 repack_chain2=1 design_chain1=0
	design_chain2=0 interface_distance_cutoff=10/>
```

```
		<ReadResfile name=resfile filename="design.resfile"/>
	</TASKOPERATIONS>
	 <FILTERS>
		<Ddg name=ddG scorefxn=score12 threshold=-20 repeats=2/>
		<Sasa name=sasa threshold=2000/>
		<CompoundStatement name=ddg_sasa>
			<AND filter_name=ddG/>
			<AND filter_name=sasa/>
		</CompoundStatement>
	 </FILTERS>
	 <MOVERS>
		<BackrubDD name=backrub partner1=0 partner2=1 interface_distance_cutoff=8.0
	moves=1000 sc_move_probability=0.25 scorefxn=score12 small_move_probability=0.15
	bbg_move_probability=0.25 task_operations=pio/>
		<RepackMinimize name=des1 scorefxn_repack=soft_rep scorefxn_minimize=soft_rep
	minimize_bb=0 minimize_rb=1 task_operations=resfile/>
		<RepackMinimize name=des2 scorefxn_repack=score12 scorefxn_minimize=score12
	minimize_bb=0 minimize_rb=1 task_operations=resfile/> Design & minimization at the interface
		<RepackMinimize name=des3 minimize_bb=1 minimize_rb=0 task_operations=resfile/>
		<ParsedProtocol name=design>
			<Add mover_name=backrub/>
			<Add mover_name=des1/>
			<Add mover_name=des2/>
			<Add mover_name=des3 filter_name=ddg_sasa/>
		</ParsedProtocol>
		<GenericMonteCarlo name=iterate scorefxn_name=score12 mover_name=design
	trials=3/>
		<InterfaceAnalyzerMover name=IAM scorefxn=score12 packstat=1 interface_sc=1
	pack_input=1 pack_separated=1 tracer=0 fixedchains=H,L/>
	</MOVERS>
	<PROTOCOLS>
		<Add mover=iterate/>
		<Add mover=IAM/>
	</PROTOCOLS>
</dock_design >
```
